# *bric à brac (bab)*, a central player in the gene regulatory network that mediates thermal plasticity of pigmentation in *Drosophila melanogaster*

**DOI:** 10.1371/journal.pgen.1007573

**Published:** 2018-08-01

**Authors:** Sandra De Castro, Frédérique Peronnet, Jean-François Gilles, Emmanuèle Mouchel-Vielh, Jean-Michel Gibert

**Affiliations:** 1 Sorbonne Université, CNRS, Laboratoire de Biologie du Développement -Institut de Biologie Paris Seine (LBD-IBPS), Team “Epigenetic control of developmental homeostasis and plasticity”, Paris, France; 2 Sorbonne Université, CNRS, Core facility, Institut de Biologie Paris Seine (IBPS), Paris, France; University of California Davis, UNITED STATES

## Abstract

*Drosophila* body pigmentation has emerged as a major Evo-Devo model. Using two *Drosophila melanogaster* lines, *Dark* and *Pale*, selected from a natural population, we analyse here the interaction between genetic variation and environmental factors to produce this complex trait. Indeed, pigmentation varies with genotype in natural populations and is sensitive to temperature during development. We demonstrate that the *bric à brac (bab)* genes, that are differentially expressed between the two lines and whose expression levels vary with temperature, participate in the pigmentation difference between the *Dark* and *Pale* lines. The two lines differ in a *bab* regulatory sequence, the dimorphic element (called here *bDE*). Both *bDE* alleles are temperature-sensitive, but the activity of the *bDE* allele from the *Dark* line is lower than that of the *bDE* allele from the *Pale* line. Our results suggest that this difference could partly be due to differential regulation by AbdB. *bab* has been previously reported to be a repressor of abdominal pigmentation. We show here that one of its targets in this process is the pigmentation gene *tan (t)*, regulated *via* the *tan* abdominal enhancer (*t_MSE*). Furthermore, *t* expression is strongly modulated by temperature in the two lines. Thus, temperature sensitivity of *t* expression is at least partly a consequence of *bab* thermal transcriptional plasticity. We therefore propose that a gene regulatory network integrating both genetic variation and temperature sensitivity modulates female abdominal pigmentation. Interestingly, both *bDE* and *t_MSE* were previously shown to have been recurrently involved in abdominal pigmentation evolution in drosophilids. We propose that the environmental sensitivity of these enhancers has turned them into evolutionary hotspots.

## Author summary

Complex traits such as size or disease susceptibility are typically modulated by both genetic variation and environmental conditions. Model organisms such as fruit flies (*Drosophila*) are particularly appropriate to analyse the interactions between genetic variation and environmental factors during the development of complex phenotypes. Natural populations carry high genetic variation and can be grown in controlled conditions in the laboratory. Here, we use *Drosophila melanogaster* female abdominal pigmentation, which is both genetically variable and modulated by the environment (temperature) to dissect this kind of interaction. We show that the pigmentation difference between two inbred fly lines is caused by genetic variation in an enhancer of the *bab* locus, which encodes two transcription factors controlling abdominal pigmentation. Indeed, this enhancer drives differential expression between the two lines. Interestingly, this enhancer is sensitive to temperature in both lines. We show that the effect of *bab* on pigmentation is mediated by the pigmentation gene *tan (t)* that is repressed by *bab*. Thus, the previously reported temperature-sensitive expression of *t* is a direct consequence of *bab* transcriptional plasticity.

## Introduction

Complex traits such as size or disease susceptibility are typically modulated by both genetic variation and environmental parameters. This has major implications in agronomy, animal husbandry and medicine. Furthermore, as the phenotype (and not the genotype) is the target of natural selection, both genetic and environmental factors are fundamental to understand evolution. Indeed, Waddington showed that a phenotype initially induced by environmental conditions can be selected and become independent of the environment [1,2]. He proposed that genetic variation present in the population was the base of this process that he termed genetic assimilation [1–3]. The importance of standing genetic variation was demonstrated by Bateman who repeated some of Waddington’s experiments and showed that genetic assimilation worked with outbred stocks but not with isogenic stocks [4]. However, in a recent study, it was shown that *de novo* mutations induced by stressful environments—such as heat-shock—can, in some cases, contribute to genetic assimilation [5]. Based on Waddington’s genetic assimilation, West-Eberhard proposed that divergent lineages could be produced through genetic assimilation of alternative morphs present in a phenotypically plastic ancestral species (“*the flexible stem hypothesis”*) [6]. A few studies suggest, indeed, that this mode of evolution, also called “*plasticity-first evolution*“, may not be uncommon in nature. For example, the tadpoles of many spadefoot toad species adjust their development time to the duration of the pond in which they live. Phylogenetic analyses show that this plasticity is ancestral. In contrast, the species *Scaphiopus couchii*, developing in ephemeral ponds, has evolved a derived and canalized short developmental time independent of the duration of the pond in which it develops (reviewed in [7]). Similarly, some generalist cichlid species show morphological plasticity of their pharyngeal jawbones in response to diet hardness. The plasticity observed in generalist species is ancestral. In contrast, other species of cichlids, which have become specialised on hard or soft diet, show only one type of morphology [8]. It is therefore important to investigate such cases at the genetic and molecular levels to understand the mechanisms of the *"flexible stem hypothesis"/"plasticity-first evolution"*. Model organisms such as *Drosophila* are particularly appropriate to dissect the interactions between genetic and environmental factors. Indeed, natural populations carry high genetic variation. Furthermore, they can be easily grown in controlled conditions in the laboratory and many genetic tools are available.

In this study, we focus on abdominal pigmentation of *Drosophila melanogaster* (*D*. *melanogaster*) females, a trait highly variable in natural populations and modulated by environmental factors such as nutrition and temperature [9–11]. Female abdominal pigmentation is darker at low temperature, in particular in the most posterior segments A5, A6 and A7 [12]. Abdominal pigmentation is therefore an example of phenotypic plasticity defined as “*the property of a given genotype to produce different phenotypes in response to distinct environmental conditions*” [13]. Lastly, as abdominal pigmentation in drosophilids is widely used as a model of intra- and inter-specific evolution [14,15], it is particularly appropriate to analyse the contribution of genetic and environmental factors to phenotypic variation and evolution.

Genome-wide association studies have identified several loci linked to female abdominal pigmentation variation in natural populations of *D*. *melanogaster* [9,11,16]. Interestingly, although the same loci were identified in different populations, their prevalence depends on the population [9,16]. In European populations, the single nucleotide polymorphisms (SNPs) that are the most strongly associated with pigmentation variation are located in the *t_MSE*, an abdominal enhancer of the *tan (t)* gene encoding an enzyme involved in melanin production [9,17]. Functional analysis of these SNPs in transgenic lines confirmed that they affect female abdominal pigmentation [18]. By contrast, in South African populations, the most significant SNPs are located in an intron of *bric à brac 1 (bab1)* [16]. *bab1* and its tandem duplicated paralogue *bab2* (named collectively *bab*) encode transcription factors involved in repression of abdominal pigmentation [19,20]. Independent genome-wide association studies performed on a North American population also identified SNPs located in the *t_MSE* and the *bab1* intron as the most significantly associated with female abdominal pigmentation variation [11]. Furthermore, in another study, genetic variation was detected in a *bab1* and *bab2 cis*-regulatory sequence, the *bab* dimorphic element (named thereafter *bDE*), which is located in the first intron of *bab1* and controls sex-specific expression of *bab1* and *bab2* in the posterior abdominal epidermis [21]. This genetic variation was associated with changes in the *bDE* activity as well as in female abdominal pigmentation [10].

Interestingly, the genes involved in female abdominal pigmentation variation are also involved in female abdominal pigmentation thermal plasticity. We previously showed that female abdominal pigmentation plasticity was caused by temperature sensitivity of a genetic network including the *bab* locus [22]. More recently, we showed that temperature modulates the expression of *t* and *yellow* (*y*), another pigmentation enzyme gene, thus contributing to female abdominal pigmentation plasticity [23,24]. The effect of temperature on *t* expression is mediated at least partly by the *t_MSE* [23]. Thus, *t* is an essential effector of female abdominal pigmentation plasticity. However, it is not excluded that temperature affects the activity or the expression of upstream regulator(s) of *t* and consequently its expression.

As the effects of genetic variation and temperature on female abdominal pigmentation were mainly studied independently, our aim was to investigate how they interact in the production of this phenotype. In this study, using two *D*. *melanogaster* lines differing in abdominal pigmentation (named thereafter *Pale* and *Dark*), we first show that genetic variation at the *bab* locus affects *bab* expression and female abdominal pigmentation. We demonstrate that the difference in *bab* expression is caused at least partly by genetic variation in the *bDE*. Indeed, a deletion that removes two binding sites for Abdominal B (Abd-B), a direct activator of *bab*, is present in the *bDE* of the *Dark* line. We show that this deletion impacts the activation of the *bDE* by Abd-B. Furthermore, the expression of *bab* is modulated by temperature in both lines, and this modulation results at least partly from temperature sensitivity of *bDE*. Lastly, *t*, whose expression is modulated by temperature, is also differentially expressed between the two lines and is repressed by *bab*. Hence, temperature modulation of *t* expression is at least partly due to *bab* transcriptional plasticity.

## Results

### The *Dark* and *Pale* lines differ in abdominal pigmentation and its thermal plasticity

**Fig 1:**
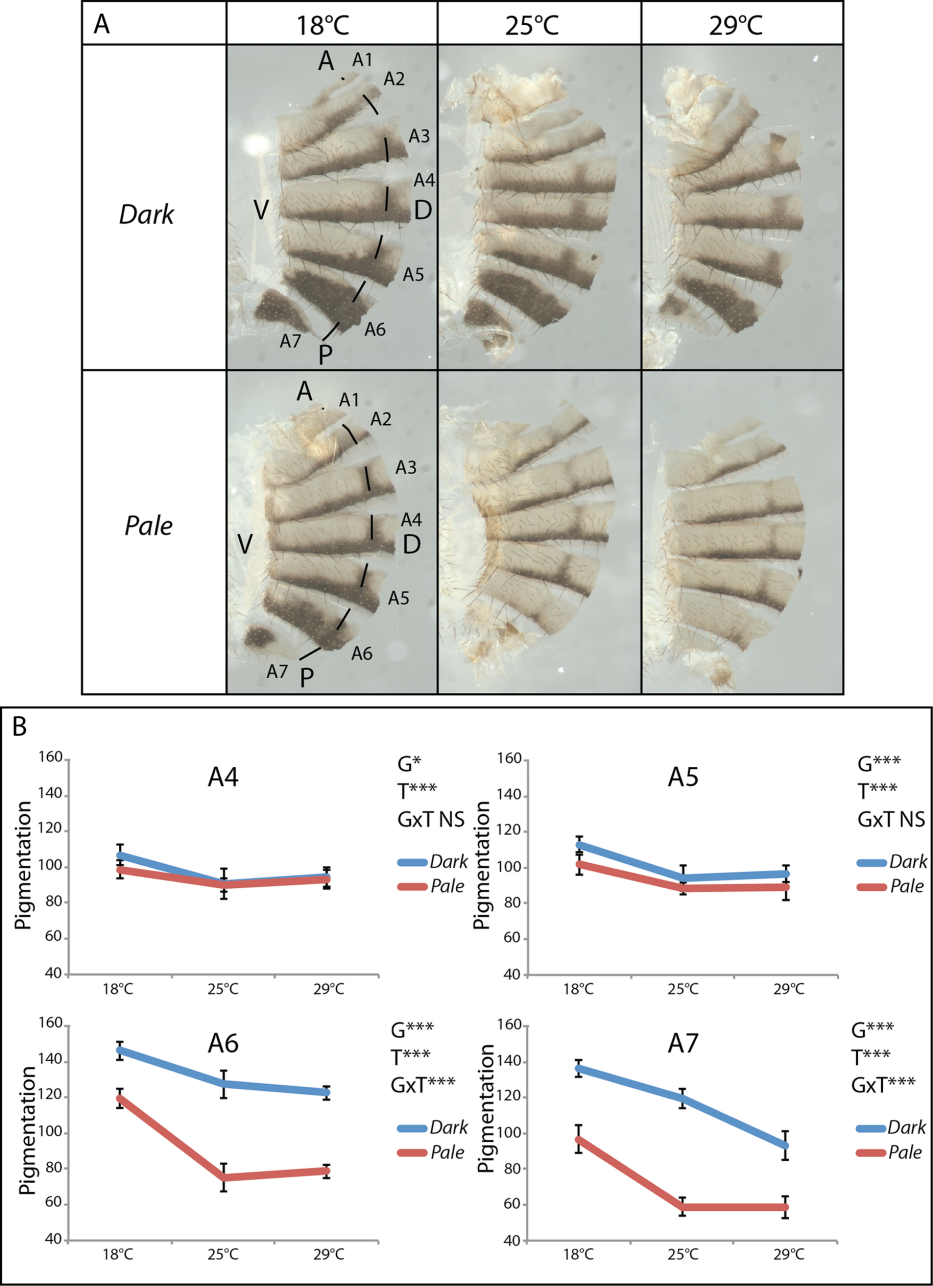
The *Dark* and *Pale* females differ in abdominal pigmentation intensity and plasticity. A: Abdominal cuticles of females from the *Dark* (up) and the *Pale* (bottom) lines grown at 18°C, 25°C or 29°C. Cuticles were cut just beyond the dorsal midline (dashed line). Hemi-abdomens are shown. Abdominal tergites A1 to A7 are indicated. A: anterior; P: posterior; V: ventral; D: dorsal. B: Reaction norms of pigmentation as a function of temperature in hemi-tergites A4, A5, A6 and A7 for the *Dark* (blue) and the *Pale* (red) lines. Error bars are standard deviations (n = 10 *per* genotype and *per* temperature). Significant effects revealed by two-way ANOVA are indicated: G = effect of the genotype, T = effect of the temperature, GxT = effect of the interaction between the genotype and the temperature. *: 0.01<p<0.05; ***: p<0.001; NS: non significant.

We established the isogenic *Pal*e and *Dark* lines at 25°C (see the material and methods section) from a natural population sampled in Canada [25] that was polymorphic for female abdominal pigmentation. To compare their abdominal pigmentation and its thermal plasticity, these lines were grown at 18°C, 25°C and 29°C. Abdominal pigmentation of A4, A5, A6 and A7 segments was quantified and analysed in females (Fig 1, S1 Dataset, Table 1 and S1 Fig). *Dark* females were darker than *Pale* females at the three temperatures. Although the effect of the genotype ("G" effect) was significant for the four segments, it was particularly pronounced in segments A6 and A7, as revealed by Eta squared (h^2^) values (A6 and A7: p<0.001, h^2^ = 0.59; A5: p<0.001, h^2^ = 0.17; A4: p<0.05, h^2^ = 0.04) (Table 1). The effect of temperature ("T" effect) was strong for both lines in all four segments (p<0.001, 0.32<h^2^<0.53). Lastly, the interaction between the genotype and the temperature ("GxT" effect) was significant for segments A6 and A7 (p<0.001). In conclusion, the *Dark* and *Pale* lines differed in pigmentation. They were both plastic in response to temperature but their plasticities differed only in segments A6 and A7.

**Table 1:** Two-way ANOVA testing the effect of the genotype (G), the temperature (T) and the genotype by temperature interaction (GxT) on pigmentation in A4, A5, A6 and A7 segments in *Dark* and *Pale* females.

	A4		A5		A6		A7	
	p	h^2^	p	h^2^	p	h^2^	p	h^2^
G	0.031	0.04	<0.001	0.17	<0.001	0.59	<0.001	0.59
T	<0.001	0.43	<0.001	0.53	<0.001	0.32	<0.001	0.33
GxT	0.090	0.04	0.27	0.014	<0.001	0.04	<0.001	0.04

p: p-value; h^2^: Eta squared.

### Difference of pigmentation between the *Dark* and *Pale* lines is linked to the *bab* locus

**Fig 2:**
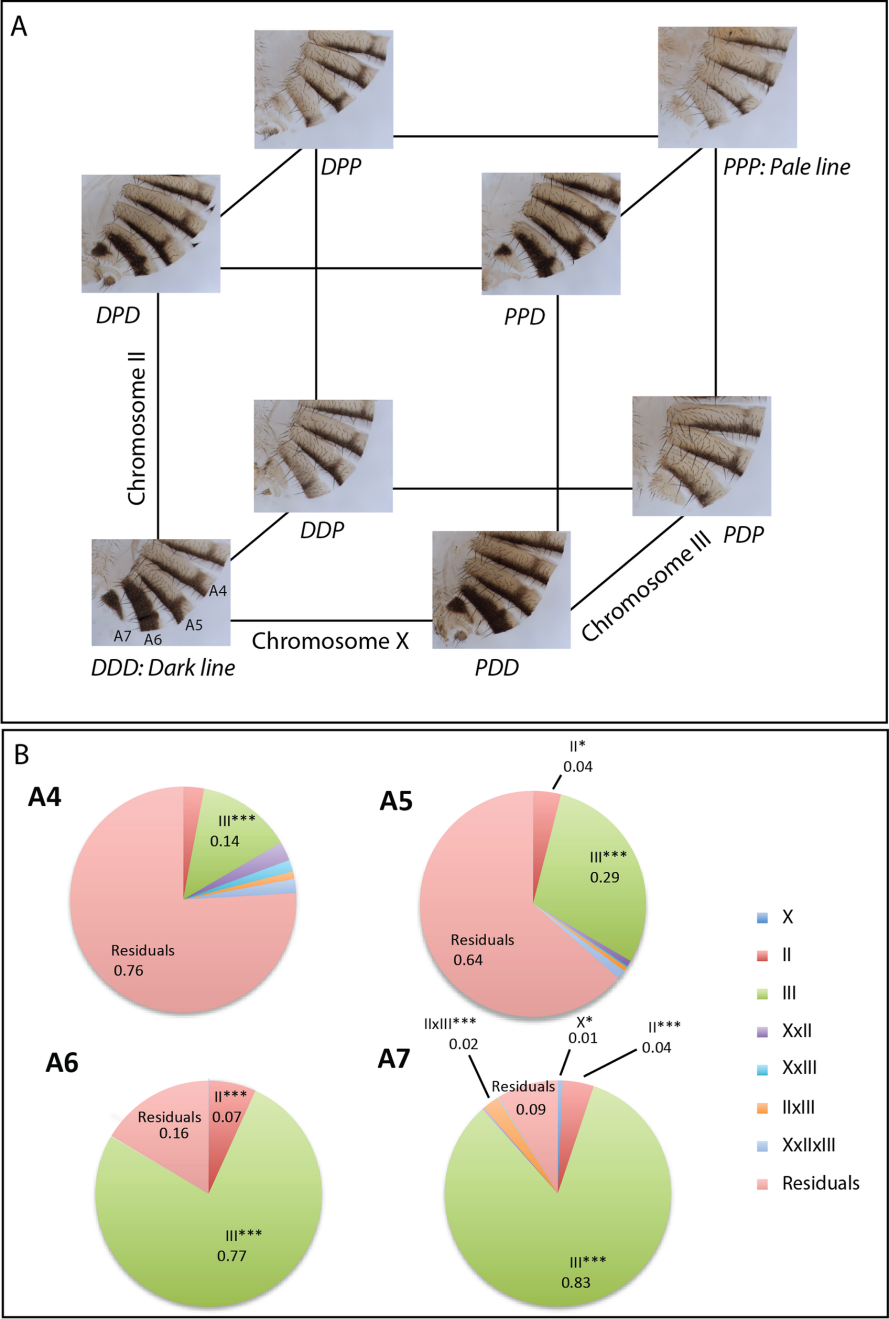
Chromosome III accounts for most of the difference of pigmentation between the *Dark* and *Pale* lines. A: Cuticles of flies, raised at 25°C, bearing the 8 combinations of X, second and third chromosomes from the *Dark* and *Pale* lines. Hemi-tergites A4 to A7 are shown. The horizontal axis represents the X chromosome, the vertical axis the second chromosome, and the oblique axis the third chromosome. Each line is named from the chromosome it contains (for example, *DPD*: X and third chromosome from the *Dark* line, second chromosome from the *Pale* line). By eye, a strong effect of the third chromosome is noticeable. B: Pie charts of the Eta squared values (h^2^) obtained from quantification of pigmentation in the 8 lines (n = 10 *per* line). The effects of each chromosome and their interactions are shown for A4, A5, A6 and A7 segments. When the effects are significant, Eta squared values are indicated on the pie. Residuals: variation not accounted by the model. *: 0.01<p<0.05; ***: p<0.001. Statistical test: three-way ANOVA with full factorial model.

We first aimed at identifying the respective contribution of each chromosome to the difference of abdominal pigmentation between the two lines. We therefore constructed new lines carrying the eight different combinations of the X, the second and the third chromosomes from the *Pale* and the *Dark* lines. We compared female abdominal pigmentation of the eight different lines grown at 25°C (Fig 2, S2 Dataset, and S2 Fig). At a glance, an effect of the third chromosome on pigmentation was noticeable for segments A4, A5, A6 and A7 (Fig 2A). Quantification and calculation of Eta squared values (h^2^) confirmed this effect of the third chromosome, which was moderate in A4 (p = 0.001, h^2^ = 0.14), strong in A5 (p<0.001, h^2^ = 0.29) and very strong in A6 and A7 (p<0.001 for both segments, h^2^ = 0.77 and 0.83, respectively) (Fig 2B and S2 Fig). In addition, the second chromosome also had a significant but much weaker effect in all segments but A4. Weak but significant effects were also observed in A7 for the X chromosome and for the interaction between chromosome II and chromosome III (Fig 2B and S2 Fig). In conclusion, these results show that one or several loci located on the third chromosome should carry genetic variation causing most of the abdominal pigmentation difference between the *Dark* and *Pale* lines. A very good candidate on this chromosome was the *bab* locus, previously identified as a major contributor for variation of female abdominal pigmentation in natural populations of *D*. *melanogaster* [10,11,16,26].

**Fig 3:**
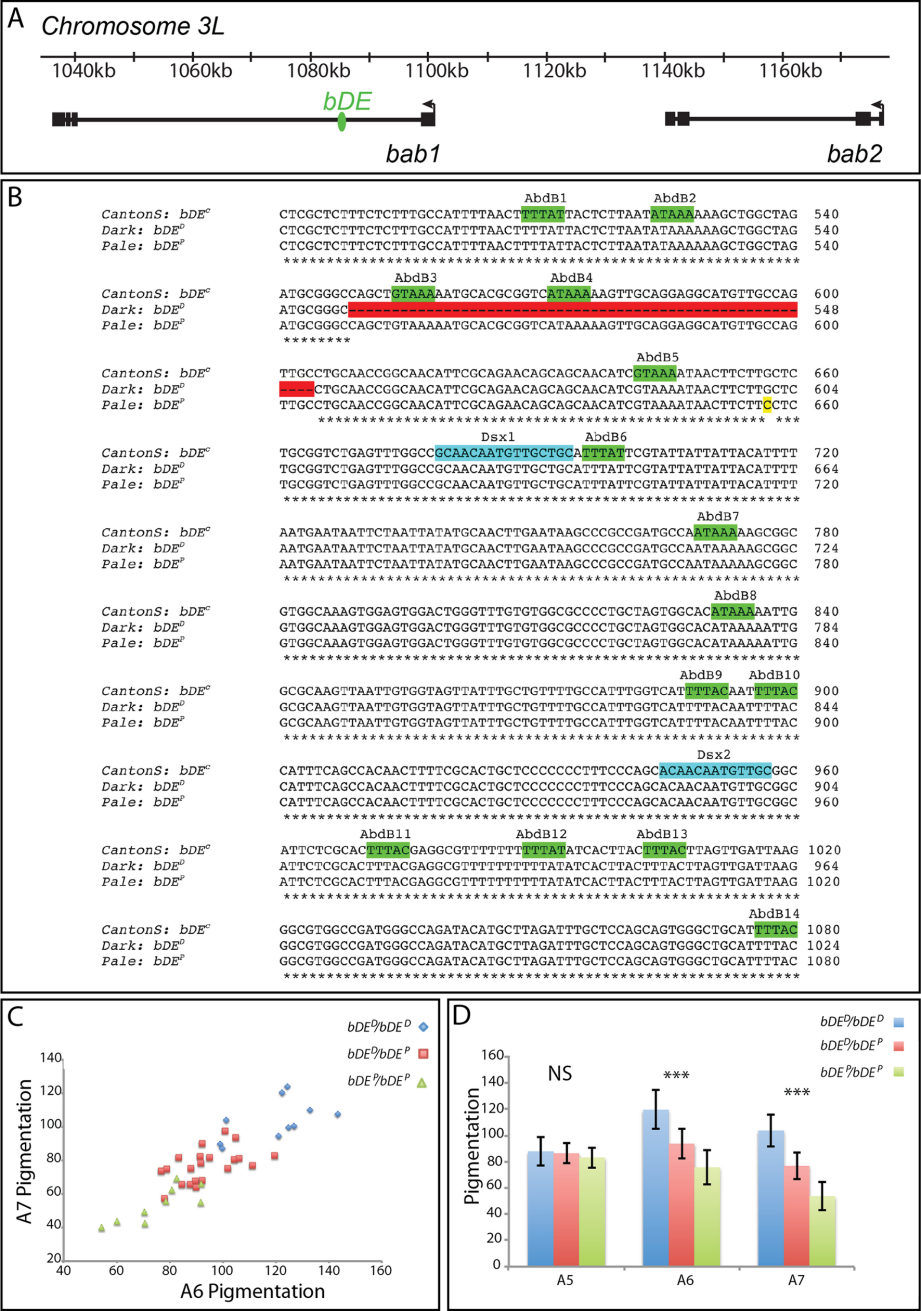
Pigmentation difference between the *Dark* and *Pale* lines is mainly linked to the *bab* locus. A: Schematic representation of the *bric à brac* locus of *D*. *melanogaster* containing the two paralogues *bab1* and *bab2*. Rectangles indicate exons and lines introns. The transcription start sites of *bab1* and *bab2* are marked by arrows. The *bab* dimorphic element (*bDE*, in green, [21]) is located in the first intron of *bab1*. B: Sequence of the *bab* dimorphic element (*bDE*) from the *Canton S* [21] (*bDE*^*C*^ allele), *Dark* (*bDE*^*D*^ allele) and *Pale* (*bDE*^*P*^ allele) lines. Binding sites for Abdominal-B (Abd-B) and Doublesex (Dsx) previously identified [21] are indicated on the *Canton S* sequence in green and blue, respectively. The haplotypes of the *Dark* and *Pale* lines differ from that of *Canton S* by a 56 bp deletion (red) and a single bp substitution (yellow), respectively. C: Scatter plot for pigmentation in A6 and A7 of 40 individuals among the F2 progeny of an initial *Dark* x *Pale* cross performed at 25°C, that were genotyped at the *bab* locus. The three genotypes clearly segregate. D: Quantification of pigmentation in A5, A6 and A7 of the F2 individuals according to their genotype at the *bab* locus. In A6 and A7, pigmentation differs between the three genotypes, which is not the case in A5. Statistical test: one-way ANOVA. ***: p<0.001. NS: non significant.

To assess a potential effect of genetic variation at the *bab* locus, we performed an association study on the F2 progeny from a cross between *Dark* females and *Pale* males. We focused on the *bab* regulatory element called *bab* dimorphic element (*bDE* [21]), located in *bab1* large intron (Fig 3A), as this enhancer was previously shown to carry high natural genetic variation with effect on female abdominal pigmentation [10]. In comparison with the *CantonS* reference haplotype (*bDE*^*C*^) [21], the *Pale* line haplotype (*bDE*^*P*^) differed by only one nucleotide (Fig 3B, yellow). In contrast, the *Dark* line haplotype (*bDE*^*D*^) presented a 56 bp deletion (Fig 3B, red), which had not been reported in previously described *bab* natural alleles. In order to investigate how frequent this deletion is in natural populations of *D*. *melanogaster*, we first analysed this region of *bDE* in the genomic sequences of 30 world-wide populations [27,28] (966 sequences in total). We never identified the 56 bp deletion in these populations. However, as this dataset of world-wide populations is focused mainly on SNPs and has not been evaluated for indels [27], it might not be optimal to identify the 56 bp deletion. Therefore, we analyzed in detail the region of the 56 bp deletion in 205 lines also originating from North America (DGRP lines from Raleigh) [29,30] using the available web-interface (http://dgrp2.gnets.ncsu.edu/). Not only SNPs but also small and large indels have been analyzed in these lines [30]. The 56 bp deletion found in the *Dark* line *bDE* was not present in any of these lines. Indeed, a SNP has been identified within the 56 bp (nucleotide 1,084,899, which corresponds to the 18^th^ position in the 56 bp sequence) and characterized in each of the 205 DGRP lines. Thus, the 56 bp deletion is likely to be a recent and rare allele, perhaps deleterious, present at low frequency at least in some Canadian populations. Although this deletion may not be relevant for adaptation, it represents an experimentally tractable system for studying the genetic and environmental interactions that affect a complex phenotype. We used this 56 bp deletion to genotype by PCR 40 F2 females randomly collected (S3 Fig). For each of them, DNA was extracted from the head and thorax whereas the abdomen was kept for pigmentation quantification. Scatter plot of pigmentation in A6 and A7 segments showed a clear segregation of the different genotypes, with *bDE*^*D*^*/bDE*^*D*^ females being darker than *bDE*^*P*^*/bDE*^*P*^ females and *bDE*^*D*^*/bDE*^*P*^ being intermediary between the two former genotypes (Fig 3C and S3 Dataset). Female abdominal pigmentation was strongly associated with the genotype at the *bab* locus in A6 (p<0.001, h^2^ = 0,61) and A7 (p<0.001, h^2^ = 0.74) but not in A5 (p = 0,49) (Fig 3D and S4 Fig). In both A6 and A7, pigmentation differs between all three genotypes (Tukey HSD test, p<0.01 for all pairwise comparisons).

In conclusion, these results strongly suggested that abdominal pigmentation variation in A6 and A7 between the *Pale* and the *Dark* lines was mainly linked to genetic variation at the *bab* locus.

### *bDE*^*D*^ is less active than *bDE*^*P*^ but both alleles are temperature-sensitive

**Fig 4:**
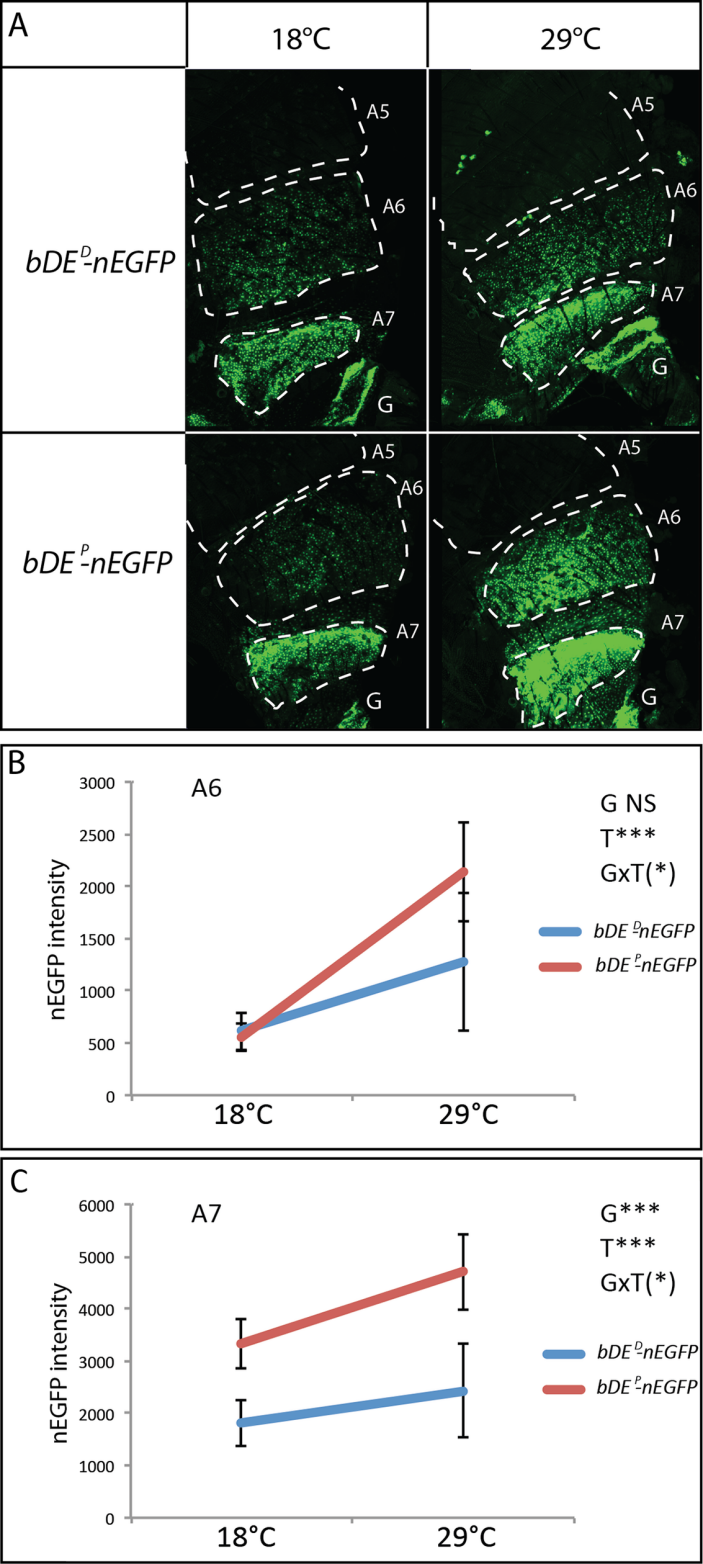
The *bDE* activity of the *Dark* and the *Pale* line is different and modulated by temperature. A: Activity of *bDE* from the *Dark* or *Pale* lines at 18°C or 29°C in young female pupae (*bDE*^*D*^*-nEGFP* and *bDE*^*P*^*-nEGFP* homozygous transgenic lines), visualized through nEGFP intensity. Segments A5, A6 and A7 are delimited with dashed lines. G: genitalia. B: Quantification of nEGFP in A6 (n = 10 *per* genotype and *per* temperature) showing a significant effect of the temperature (T) and a marginally significant effect of the genotype by temperature interaction (GxT). Non-parametric ANOVA (Sheirer-Ray-Hare test). (*) 0.05<p<0.1; ***: p<0.001. NS: non significant. C: Quantification of nEGFP in A7 (n = 10 *per* genotype and *per* temperature) showing significant effects of the genotype (G) and the temperature (T). A marginally significant effect of the genotype by temperature interaction (GxT) is also observed. Statistical test: two-way ANOVA. (*): 0.05<p<0.1; ***: p<0.001.

The *bDE* is directly activated by the Doublesex female-specific isoform (Dsx^F^) and the Hox protein Abdominal B (Abd-B) in the abdominal epidermis of females [27]. Interestingly, the 56 bp deletion present in the *bDE*^*D*^ allele of the *Dark* line, removes two Abd-B binding sites (Fig 3B). These sites are the sites Abd-B3 and Abd-B4 that were shown to bind Abd-B *in vitro* and to contribute, together with twelve other Abd-B binding sites, to *bDE* activity *in vivo* [21]. The *bDE*^*P*^ allele of the *Pale* line also differs from *bDE*^*C*^ and *bDE*^*D*^ by a single nucleotide substitution (Fig 3B, yellow). This SNP was previously reported to reduce the activity of the enhancer [10]. To test whether the 56 bp deletion of the *bDE*^*D*^ allele could also affect the activity of the enhancer, we constructed transgenic lines in which nuclear enhanced green fluorescent protein (nEGFP) was under the control of *bDE*^*D*^ or *bDE*^*P*^ (lines *bDE*^*D*^*-nEGFP* and *bDE*^*P*^*-nEGFP*). In order to compare the activities of these two variants, the transgenes were inserted at the same genomic location using *phiC31*-based transgenesis [31], thus avoiding position effects. Then, they were introgressed for six generations in the same genetic background. The resulting lines, homozygous for the transgenes, were grown at 18°C and at 29°C, in order to test the potential effect of temperature on *bDE* activity. *bDE* activity was previously shown to peak in the abdominal epidermis from female pupae [21]. We therefore quantified nEGFP at this stage (Fig 4, S4 Dataset and S5 Fig). Both *bDE*^*D*^ and *bDE*^*P*^ drove nEGFP in A6 and A7 but not in A5 (Fig 4A), as already described for *bDE*^*C*^ [21]. In A6, the two *bDE* alleles presented a different activity at 29°C (t-test, p<0.01) but not at 18°C, which led to a non-significant effect of the genotype ("G" effect) (Figs 4B and S5). By contrast, in A7, the effect of the genotype on nEGFP expression was significant (Figs 4C and S5; "G" effect, p<0.001). Interestingly, for the two lines and in both A6 and A7, nEGFP was significantly more expressed at 29°C than at 18°C ("T" effect, p<0.001). The genotype by temperature interaction was marginally significant in both segments ("GxT" effect, p = 0.094 and p = 0.073, respectively) (Figs 4B, 4C and S5).

In conclusion, *bDE*^*D*^ was less active than *bDE*^*P*^. Moreover, temperature modulated the activity of both alleles, which were less active at 18°C than at 29°C. As *bab* represses melanin production [20], the lower activity of *bDE*^*D*^ and the higher activity of both alleles at high temperature correlates with pigmentation intensity. Since the 56 bp deletion of *bDE*^*D*^ removes two binding sites for Abd-B, which activates this enhancer, loss of these sites might participate in the reduction of activity of *bDE*^*D*^ as compared to *bDE*^*P*^.

### The deletion removing two Abd-B binding sites in *bDE*^*D*^ impacts enhancer activation by Abd-B

**Fig 5:**
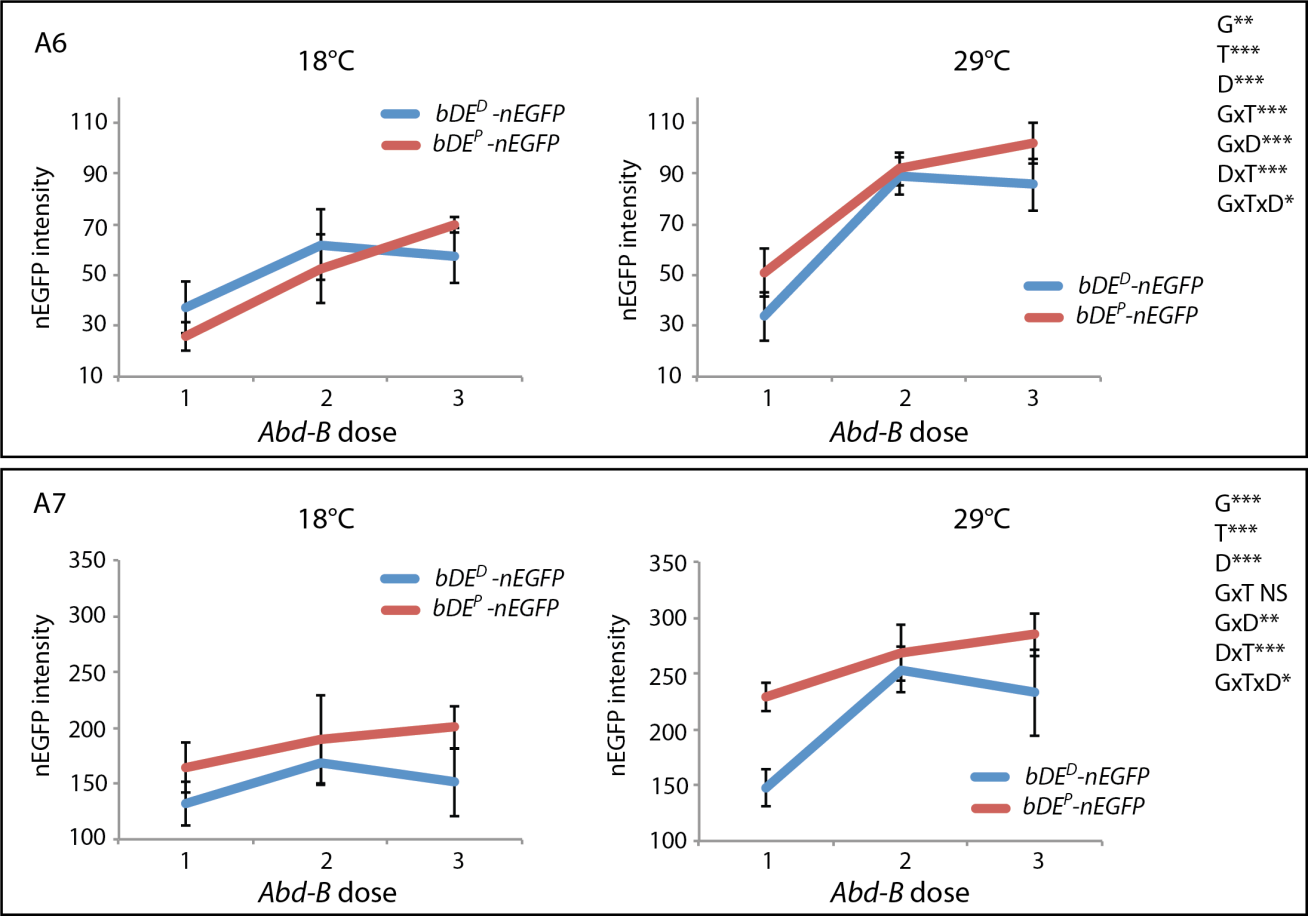
*Abd-B* dose, genotype and temperature modulate the activity of *bDE*. Quantification of nEGFP in A6 and A7 (n = 10 *per* condition) with variation of the temperature (T: 18°C or 29°C), *Abd-B* dose (D: 1, 2 or 3) and genotype (G: *bDE*^*D*^ or *bDE*^*P*^). Three-way ANOVA were performed for each segment on Box-Cox transformed measures of nEGFP intensity extracted from nEGFP positive nuclei. * p<0.05; **p<0.01; ***: p<0.001. NS: non significant.

To test the impact of the 56 bp deletion in *bDE*^*D*^ on its activation by Abd-B, we introduced chromosomes with a deletion or a duplication of *Abd-B* in the *bDE*^*D*^*-nEGFP* and *bDE*^*P*^*-nEGFP* transgenic lines. We thus obtained flies heterozygous for *bDE*^*D*^*-nEGFP* or *bDE*^*P*^*-nEGFP*, and expressing one dose, two doses or three doses of *Abd-B*. Flies were grown at 18°C or 29°C and nEGFP intensity in A6 and A7 was quantified. The different conditions of genotype, temperature and *Abd-B* dose were then compared (Fig 5, S5 Dataset, S6 and S7 Figs). As already observed with homozygous nEGFP transgenes (Fig 4), the genotype effect (“G”) as well as the temperature effect (“T”) were significant for A6 and A7 (“G” for A6: p<0.01, h^2 =^ 0.008; “G” for A7: p<0.001, h^2^ = 0.147; “T” for A6: p<0.001, h^2^ = 0.235; “T” for A7: p<0.001, h^2^ = 0.394). A significant effect of the genotype by temperature interaction was also observed for A6 ("GxT": p<0.001, h^2^ = 0.021). These results confirmed that the activity of *bDE* depended on the allele and on the temperature, and that the effect of temperature was modulated by the genotype.

The effect of *Abd-B* dose was strong and significant for A6 and A7 (“D” for A6: p<0.001, h^2^ = 0.540; “D” for A7: p<0.001, h^2^ = 0.193). This result was expected as Abd-B was shown to be a direct activator of *bDE* [21] and as *bDE*^*D*^ and *bDE*^*P*^ share twelve Abd-B sites. Very interestingly, a significant effect of the genotype by *Abd-B* dose interaction was observed ("GxD" for A6: p<0.001, h^2^ = 0.021; for A7: p<0.01, h^2^ = 0.024), demonstrating that the 56 bp deletion in *bDE*^*D*^ had a direct impact on the activation of this enhancer by Abd-B. This effect could be the consequence of the removal of the two Abd-B sites, the changing of spacing between remaining Abd-B sites, or the removal of other uncharacterized regulatory sites.

Furthermore, there was a significant effect of the *Abd-B* dose to temperature interaction, independently of the genotype and thus the deletion ("DxT" for A6: p<0.001, h^2^ = 0.038; for A7: p<0.001, h^2^ = 0.034,). This means that the effect of Abd-B on *bDE* activity, for both *bDE* alleles, was modulated by temperature. However, we did not detect any variation of *Abd-B* expression in the female abdominal epidermis between 18°C and 29°C, indicating that this modulation was not a consequence of *Abd-B* differential expression (S8 Fig and S6 Dataset). The effect of temperature on Abd-B thus might be post-transcriptional (activity of the protein itself, binding to the enhancer, interaction with co-factors …).

Lastly, a weak but significant interaction between genotype, temperature and *Abd-B* dose was observed, ("GxTxD" effect for A6: p<0.05, h^2^ = 0.010; for A7: p<0.05, h^2^ = 0.012), indicating that the difference in thermal plasticity between *bDE*^*D*^ and *bDE*^*P*^ was partly due to Abd-B.

### Temperature and *Abd-B* dose have an impact on *bab1* and *bab2* expression

**Fig 6:**
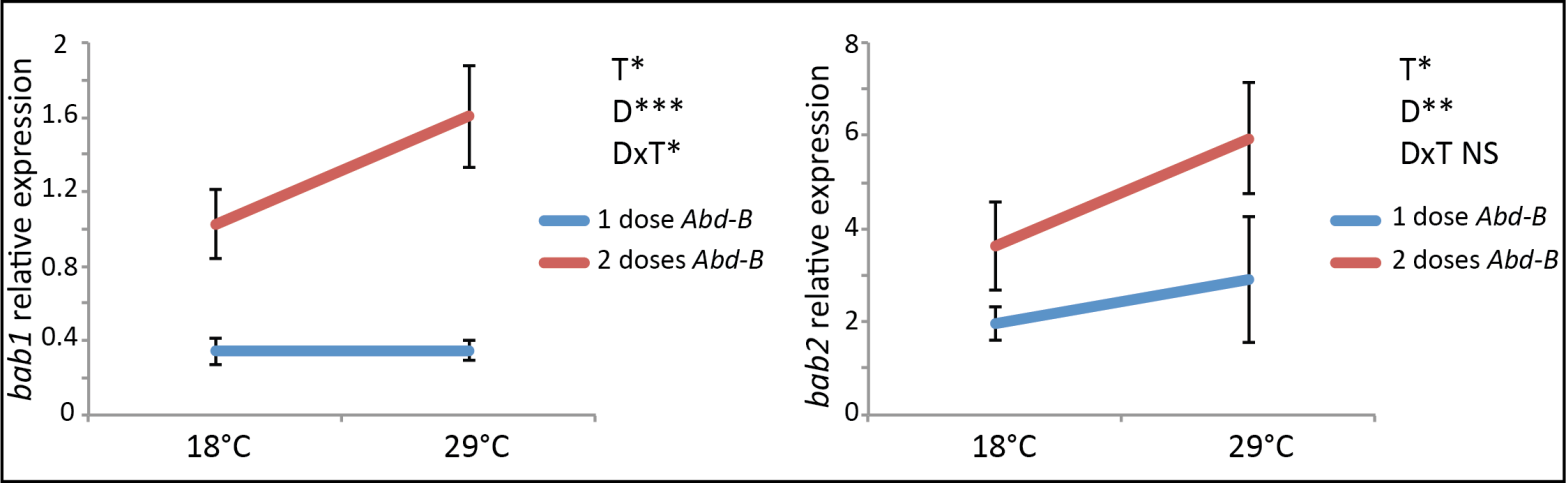
Temperature and *Abd-B* dose have an impact on *bab1* and *bab2* expression. RT-qPCR quantification of *bab1* and *bab2* expression in posterior abdominal epidermis from pupae expressing 1 or 2 doses of *Abd-B* and grown at 18°C and 29°C. Expression of *bab1* and *bab2* was normalized with the geometric mean of *eIF2* and *Spt6* expression. Error bars represent standard deviation (3 replicates of 50 individuals per condition). Statistics: two-way ANOVA. T: effect of temperature; D: effect of *Abd-B* dose. DxT: effect of the interaction between *Abd-B* dose and temperature. *: 0.01<p<0.05; **: 0.001<p<0.01; ***: p<0.001; NS: non significant.

As the effect of Abd-B on *bDE* activity was modulated by temperature independently of the *bDE* genotype (Fig 5, "DxT" effect), we wondered whether we could detect an effect of temperature and of *Abd-B* dose on *bab1* and *bab2* regulation. We thus quantified by RT-qPCR *bab1* and *bab2* expression in the posterior epidermis of female pupae grown at 18°C or 29°C and expressing one, two or three doses of *Abd-B* (Fig 6, S7 Dataset and S9 Fig). Unfortunately, we could not interpret the data for three doses of *AbdB* because the reference genes we used were themselves deregulated. A weak but significant effect of temperature was observed for *bab1* and *bab2* (“T” effect, *bab1*: p<0.05, h^2^ = 0.072; *bab2*: p<0.05, h^2^ = 0.23). Thus, temperature modulation of *bDE* activity may impact *bab1* and *bab2* expression.

A very strong effect of *Abd-B* dose was observed for both genes (“D” effect, *bab1*: p<0.001, h^2^ = 0,792; *bab2*: p<0.01, h^2^ = 0.480). This was expected as Abd-B directly activates *bDE* [21]. Lastly, a significant interaction between *Abd-B* dose and temperature was observed for *bab1* (“DxT” effect, p<0.05, h^2^ = 0.070). Thus, temperature modulates the effect of Abd-B on *bab1* expression, which suggests that the effect of temperature on *bDE* activation by Abd-B has functional consequences.

### *bab1* and *bab2* expression differs between the *Pale* and *Dark* lines and is temperature-sensitive

**Fig 7:**
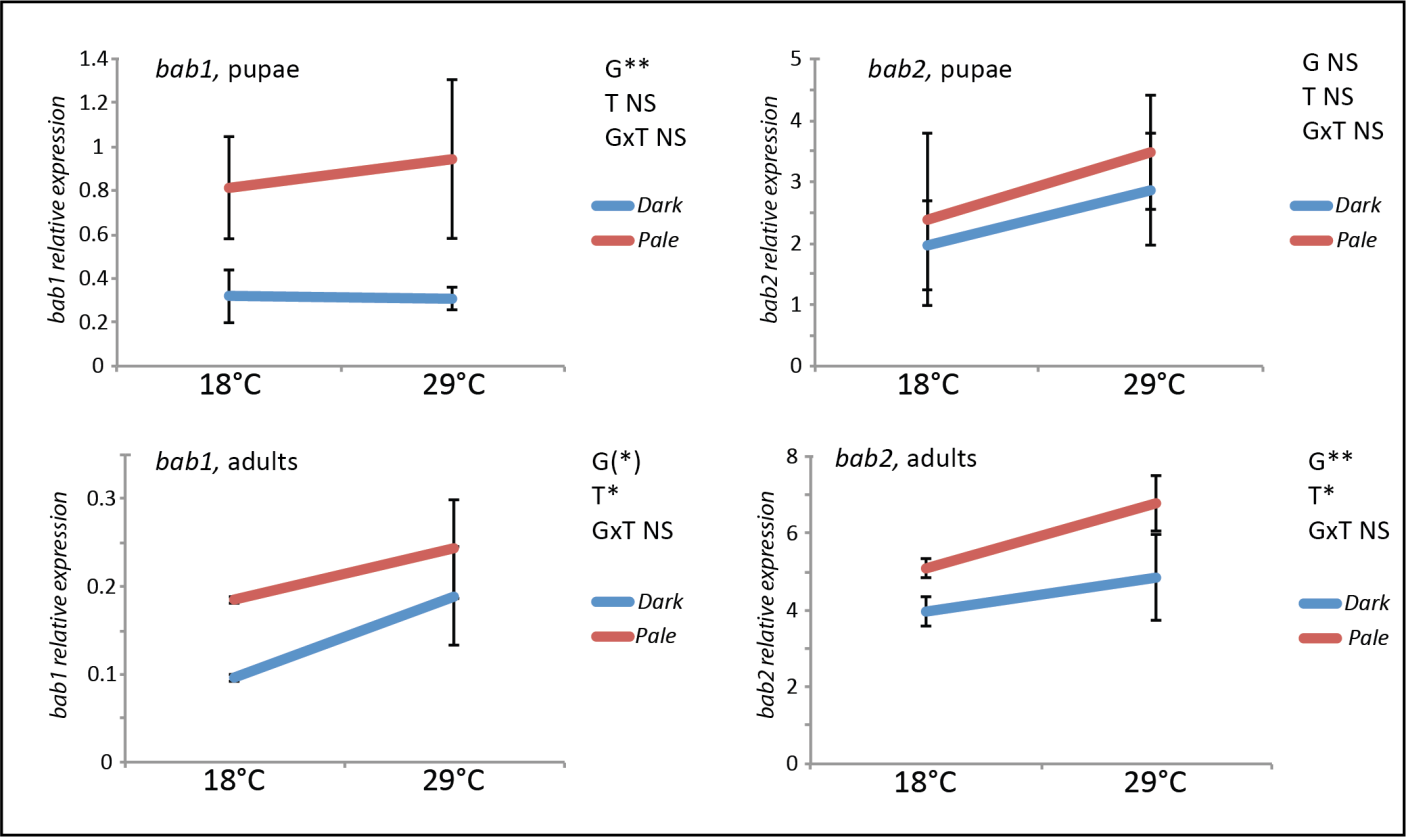
Expression of *bab 1* and *bab2* differ between the *Dark* and the *Pale* line and varies with temperature. RT-qPCR quantification of *bab1* and *bab2* expression in posterior abdominal epidermis (segments A5, A6 and A7) from pupae and young adult females of the *Dark* and *Pale* lines raised at 18°C or 29°C. Expression of *bab1* and *bab2* was normalized with the geometric mean of *eIF2* and *Spt6* expression. Error bars represent standard deviation (3 replicates of 50 individuals *per* condition). Statistics: two-way ANOVA (*bab*1 in pupae, *bab2* in pupae and adults) or non-parametric ANOVA Scheirer-Ray-Hare test (*bab1* in adults). G: effect of the genotype; T: effect of the temperature; GxT: effect of the interaction between the genotype and the temperature. (*): 0.05<p<0.1; *: 0.01<p<0.05; **: 0.001<p<0.01. NS: non significant.

In Fig 4, we showed, using reporter constructs in transgenic flies, that *bDE*^*D*^ is less active than *bDE*^*P*^ but that both alleles are temperature-sensitive. To test whether these effects could have an impact on *bab1* and *bab2* expression, we quantified by RT-qPCR *bab1* and *bab2* mRNA in the posterior abdominal epidermis of female pupae and young adults from the *Dark* and *Pale* lines (Fig 7, S8 Dataset and S10 Fig). *bab1* was less expressed in the *Dark* line than in the *Pale* line both in pupae and in adults at the two temperatures ("G" effect; pupae: p<0.01, between 2.6 and 3 times less depending on the temperature; adults: p = 0.055, between 1.3 and 1.9 times less depending on the temperature). Expression of *bab2* was also lower in the *Dark* line but significantly only in adults ("G" effect, p<0.01, between 1.3 and 1.4 times less depending on the temperature). In addition, expression of *bab1* and *bab2* was modulated by temperature in adults, as both genes were more expressed at 29°C than at 18°C ("T" effect, p<0.05, between 1.2 and 1.9 times more at 29°C than at 18°C depending on the line and the gene). Thus, the lower expression of *bab* paralogues in the *Dark* line as compared to the *Pale* line, and their higher expression at high temperature, correlated with *bDE* activity and pigmentation intensity.

### *bab* represses *t* expression in abdominal epidermis

**Fig 8:**
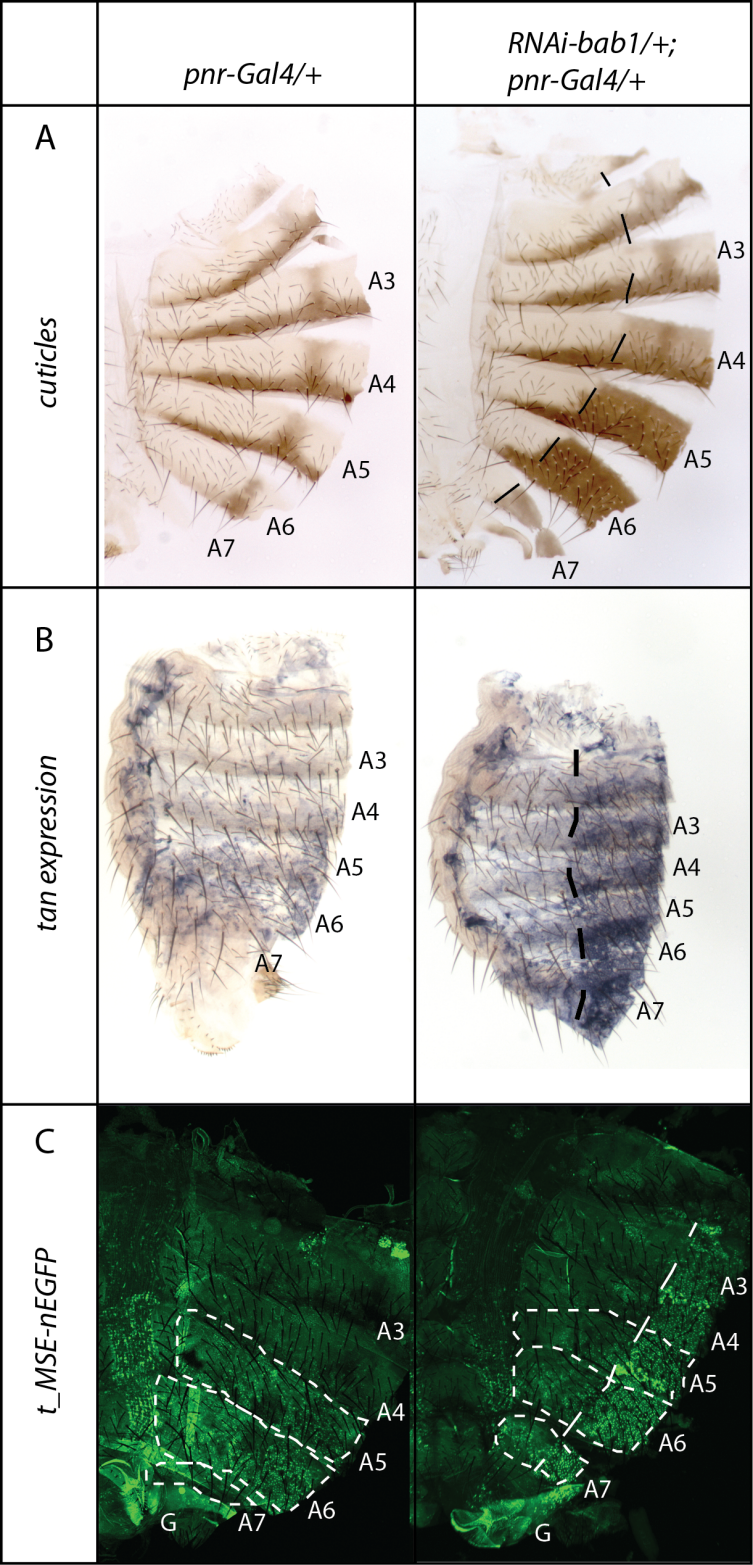
Down-regulation of *bab1* in the abdominal epidermis increases *t* expression and *t_MSE* abdominal enhancer activity. A: Adult cuticles of control female (left) and female in which *bab1* was down-regulated using a *UAS-RNAi bab1* transgene and the *pnr-Gal4* driver (right). *bab1* down-regulation induces an increase of melanin production in the *pnr-Gal4* expression domain. B. *In situ* hybridization experiments on the same genotypes as A showing *t* expression in the abdomen of freshly hatched females. Compared to the control (left), *bab1* down-regulation (right) increases *t* expression in the *pnr-Gal4* expression domain. C. Effect of *bab1* down-regulation on the activity of the *t_MSE* abdominal enhancer (*t_MSE-nEGFP* transgenic line). Compared to the control (left), *bab1* down-regulation in the *pnr-Gal4* expression domain induces an increase of nEGFP expression driven by the *t* abdominal enhancer *t_MSE* (right). G: genitalia.In A and B, crosses were performed at 29°C to maximize the difference of pigmentation between the dorsal region and the control lateral region. In C, crosses were performed at 25°C. In A, B and C, the dashed lines mark the limit between the dorsal stripe (on the right) in which *pnr-Gal4* is expressed and the lateral region (on the left) used as an internal control.

The two paralogues *bab1* and *bab2* encode transcription factors with BTB/POZ and Psq domains, which are involved in repression of abdominal pigmentation [19,20]. Among pigmentation genes, it is already known that *bab* represses *y* and *Dopa-decarboxylase* (*Ddc*) [22,32,33]. Based on previous studies showing the essential role of *t* in female abdominal pigmentation intensity and plasticity [9,18,23], we wondered whether *t* was a target of *bab* in abdominal epidermis. Indeed, this was suggested in several studies, but never demonstrated [10,14,15]. To test this hypothesis, we down-regulated *bab2* and/or *bab1* in the abdominal epidermis using *UAS-RNAi* transgenes combined with the drivers *pannier-Gal4* (*pnr-Gal4*) [34] or *yellow-Gal4* (*y-Gal4*) [35]. As expected, *bab* down-regulation increased abdominal pigmentation (Figs 8A and S11A). Furthermore, *t* expression, revealed by *in situ* hybridization, increased when *bab1* was down-regulated (Fig 8B). To confirm this result, *bab* was down-regulated in two reporter lines inducing nEGFP expression under the control of *t* regulatory sequences: the *t_MSE-nEGFP* line, that contained only the *t* abdominal enhancer *t_MSE* [36], and the *5't-nEGFP* line, that contained a genomic fragment about 4 kb long located directly upstream the *t* transcription start site and including the *t_MSE*. In these two lines, *bab* down-regulation induced an increase of *nEGFP* expression (Figs 8C and S11B).

Taken together, these results demonstrated that *t* expression was repressed by *bab*, and that this repression was mediated, at least partly, by the *t* abdominal enhancer *t_MSE*.

### *t* is differentially expressed between the *Dark* and the *Pale* lines

**Fig 9:**
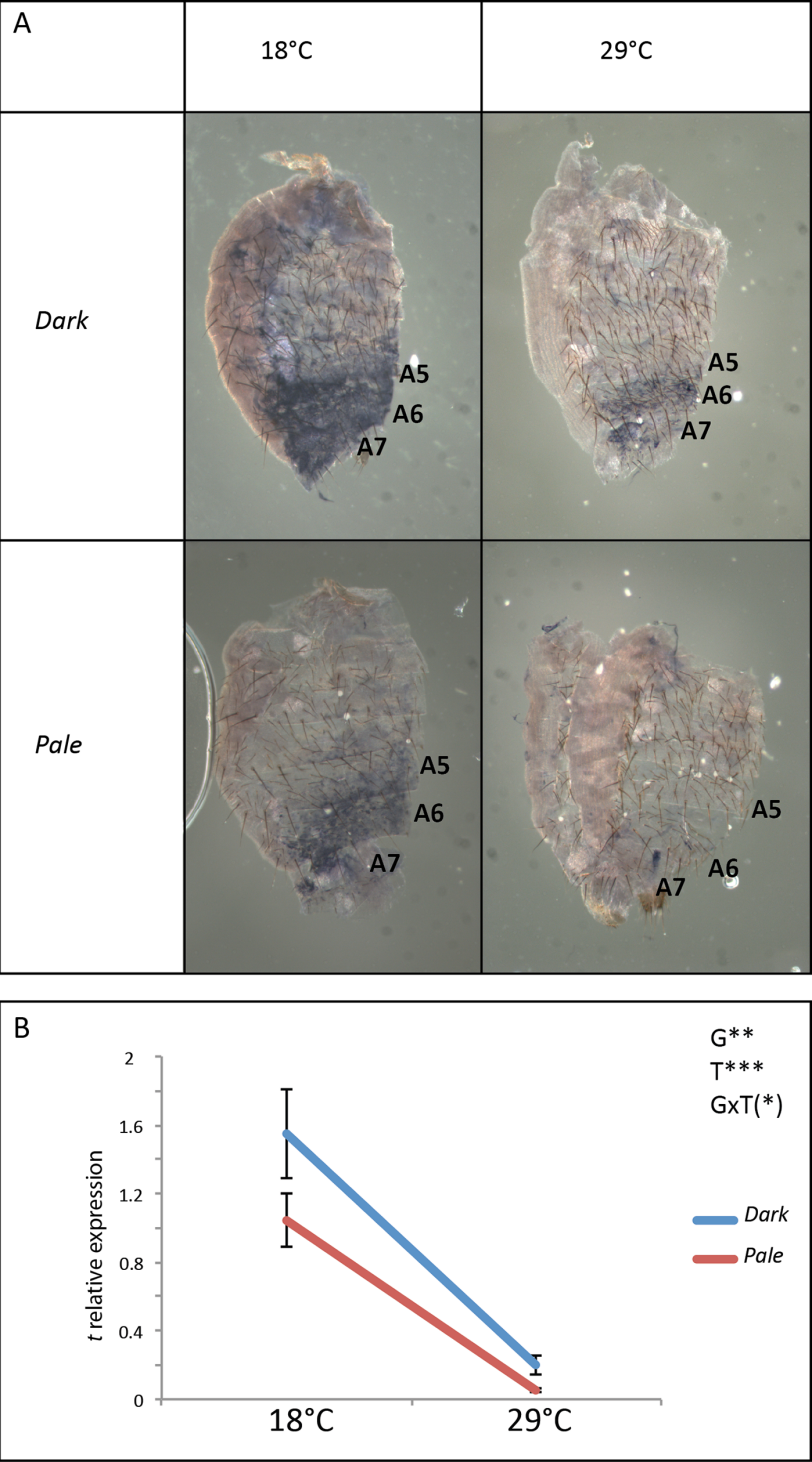
Expression of *t* is different between the *Dark* and the *Pale* lines and is modulated by temperature. A: *in situ* hybridization to reveal *t* expression in A5, A6 and A7 segments of *Dark* and *Pale* females grown at 18°C or 29°C. The dorsal side of the cuticle is on the right. B: RT-qPCR quantification of *t* expression in the posterior abdominal epidermis (A5, A6 and A7 segments) of young adult females of the *Dark* and *Pale* lines raised at 18°C or 29°C. The expression of *t* was normalized with the geometric mean of *Act5c* and *RP49* expression. Error bars represent standard deviations (3 replicates of 50 individuals *per* condition). Statistics: ANOVA. G: effect of the genotype; T: effect of the temperature; GxT: effect of the interaction between the genotype and the temperature. (*): 0.05<p<0.1; **: 0.001<p<0.01. ***: p<0.001.

As shown above, *bab* expression differs between the *Dark* and *Pale* lines and is modulated by temperature. In addition, *bab* represses *t* expression. In order to test whether *bab* differential expression in the *Pale* and *Dark* lines impacts *t* expression, we performed *in situ* hybridization experiments and RT-qPCR quantification in abdominal epidermes of young females of the two lines raised at 18°C or 29°C (Fig 9, S9 Dataset and S12 Fig). As we showed previously in another *D*. *melanogaster* line (*w*^*1118*^ line, [23]), *t* expression was strongly modulated by temperature in the two lines. Indeed, *t* was 20 times more expressed at 18°C than at 29°C in the *Pale* line, and 7.7 times in the *Dark* line ("T" effect: p<0.001, h^2^ = 0.87). There was also a significant effect of the genotype, although less strong. Indeed, at 18°C, *t* was 1.5 times more expressed in the *Dark* line than in the *Pale* line, and 3.8 times more at 29°C ("G" effect, p<0.01, h^2^ = 0.068). Lastly, interaction between the genotype and the temperature was marginally significant, indicating that their effects on *t* expression were mainly additive ("GxT" effect: p = 0.08, h^2^ = 0.021).

In conclusion, *t* was differentially expressed between the *Pale* and the *Dark* lines and modulated by temperature in both lines. As *t* was repressed by *bab*, this differential expression may result at least partly from the differential expression of *bab* between the two lines and the temperature sensitive expression of *bab* in both lines.

## Discussion

Here, we use two *Drosophila melanogaster* lines, *Dark* and *Pale*, established from a natural population, to study the regulatory mechanisms of abdominal pigmentation intensity and plasticity. We demonstrate that *bab* expression differs between the two lines. This is partly due to the existence of a deletion in the *bab* dimorphic element (*bDE*) that modulates the action of Abd-B on *bDE* activity. *bab* in turn represses *t* through the *t_MSE*. The expression of *t* differs between the *Dark* and the *Pale* lines, and this effect is mainly caused by variation in *trans* at the *bab* locus. Indeed, contribution of the X chromosome carrying *t* is non-significant or extremely weak depending on the segments. Absence of functional variation in *cis* at the *t* locus contrasts with previous results on European populations, in which SNPs in *t_MSE* accounted for most variation in female abdominal pigmentation [9]. Indeed, sequencing of *t_MSE* of the *Dark* and *Pale* lines revealed that they have exactly the same sequence (see the Material and Methods section).

**Fig 10:**
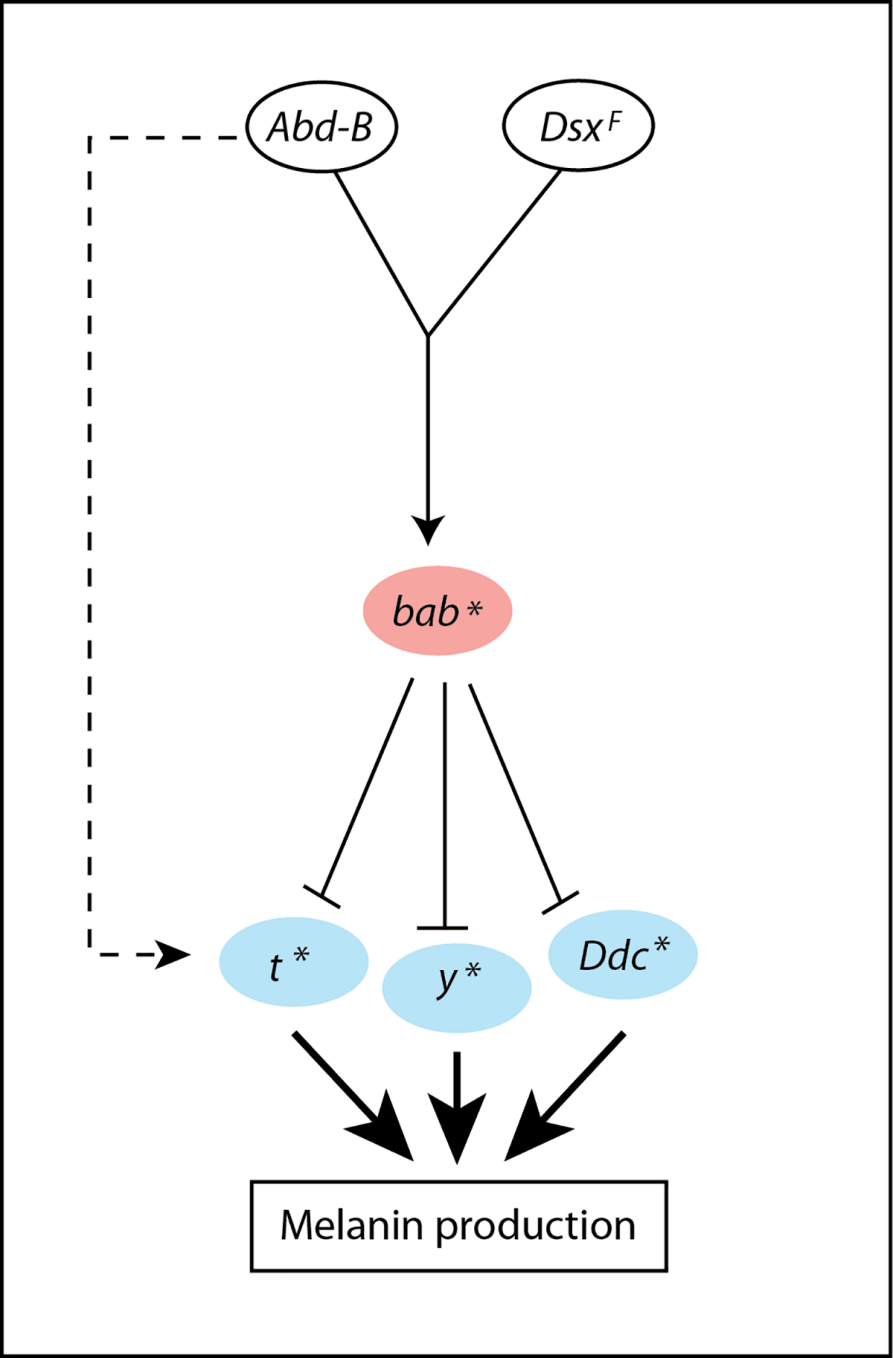
Gene regulatory network mediating the effect of temperature on female abdominal pigmentation in the posterior abdomen. In the posterior abdominal epidermis of females, *bab* is activated by *Abd-B* and *DsxF*. *bab* expression is higher at high temperature (pink), which leads to a lower expression of *t*, *Ddc* and *y* at high temperature (blue). The resulting effect is a decrease of melanin production at high temperature. *Abd-B* might activate *t* at low temperature, which would explain the high expression of *t* when *bab* expression is low. Asterisks indicate the genes for which expression in the posterior abdominal epidermis was demonstrated to be sensitive to temperature.

Our results allow also a better understanding of the effect of temperature on female abdominal pigmentation (Fig 10). *Abd-B* lies at the top of a gene network and plays an essential role in plasticity. Indeed, plasticity of pigmentation increases along the antero-posterior axis in parallel with *Abd-B* expression [12,37]. Furthermore, ectopic expression of *Abd-B* in the thorax is sufficient to generate a sex-specific highly plastic pigmentation pattern [22]. *Abd-B* has opposite effects on pigmentation depending on temperature, as it induces strong melanin production at 18°C, whereas repressing all cuticular pigments at 29°C [22]. Moreover, Abd-B and the female-specific isoform of Doublesex (Dsx^F^) activate *bab* through binding to *bDE* [21]. We show here that the impact of *Abd-B* on *bDE* activity varies with temperature, increasing expression of *bab* at high temperature. As *Abd-B* expression does not vary with temperature, this is likely to be due to post-transcriptional mechanisms. In addition, a part of the modulation by temperature of *bDE* activity and *bab* genes expression occurs independently of Abd-B (represented by the “T” effect for *bDE* activity or *bab1* and *bab2* expression). The causal mechanism is for the moment unknown. In a previous study, we showed that *bab* belongs to a chromatin regulator network that mediates the effect of temperature on female abdominal pigmentation [22]. Our new results show that transcriptional modulation of *bab* by temperature through the *bDE* is important for temperature-sensitivity of the network. Furthermore, as *bab* represses *t*, temperature-sensitive expression of *t* in female posterior abdominal epidermis, previously reported [23] and confirmed in this study, is, at least partly, a consequence of *bab* temperature-sensitive expression. Interestingly, expression of other pigmentation enzymes, such as *Ddc* or *y*, is also repressed by *bab* and sensitive to temperature [22–24,33]. This is probably caused, at least partly, by the temperature-sensitive expression of *bab*. Furthermore, the global repressive role of Abd-B on pigmentation enzymes at high temperature goes at least partly through *bab*. *y* was recently shown to be a direct target of *bab* [33]. Whether *t* and *Ddc* are direct targets of *bab* remains unknown. Lastly, the mechanism underlying the activator role of Abd-B on melanin production at low temperature is unknown but may rely on the activation of *t* and/or other pigmentation enzyme genes.

Classically, two types of genetic effects are postulated to be involved in phenotypic plasticity [38]. “*Allelic sensitivity*” applies to a gene whose expression or product activity depends on the environment. “*Gene regulation*” applies to a regulatory gene that turns on or off its targets depending on the environment. Our study illustrates how these effects may blur when considering regulatory genes and their targets. The temperature sensitivity of *bab* expression could be classified as “*allelic sensitivity*”. However, because *bab* encodes a transcriptional repressor, it corresponds also to a “*gene regulation*” effect when considering the effect on its targets. Furthermore, *bab* thermal plasticity leads to temperature-sensitive expression of *t*, another case of “*allelic sensitivity*”.

A few other gene networks mediating phenotypic plasticity have been described in other species [39,40]. Similarly to the network we describe, these networks are composed of regulatory genes (involved in hormonal pathways, transcription factors) responding to the environment and structural genes that are their downstream targets. For example, pharyngeal jaw plasticity in cichlids is mediated by a complex gene network in which the transcription factor AP1 plays a major role as it is sensitive to the mechanical strain exerted by the food [39]. AP1 regulates another transcription factor *Runx2B*, which activates the structural gene *osx*, a key osteoblast regulator [39]. Such a gene regulatory network perspective is needed to understand how phenotypic plasticity is mediated and how it could be involved in evolution by genetic assimilation [41].

Our results corroborate other studies on fly pigmentation variation and evolution which have identified changes in *cis*-regulatory sequences involved in evolution of body or wing pigmentation (reviewed in [14,15]). Indeed, genetic variation in a modular enhancer such as *bDE* affects only one trait (abdominal pigmentation) leaving unaffected other traits controlled by *bab via* distinct enhancers (legs and ovaries) [19]. Similarly, in vertebrates, it has been shown that morphological variation within or between species very often relies on genetic changes in modular enhancers and not in coding sequences. In several examples, loss of particular structures are associated with mutations reducing the activity of an enhancer: parallel pelvic plate reduction in sticklebacks is caused by recurrent deletions of an enhancer of *Pitx1* [42]; limb loss in snakes is caused by nucleotide changes in an enhancer of *Sonic hedgehog* [43,44]. Conversely, mutations increasing the activity of an enhancer are involved in the enlargement of particular structures. For example, in bats, a limb specific enhancer of *Prx1* that has a higher activity than its mouse homologue induces significantly longer forelimbs [45]. Some mutations in *cis-*regulatory sequences can also modify the timing of gene expression: persistent expression of human lactase expression in adult small intestine is linked to several mutations in the regulatory sequences of the lactase gene that have occurred independently in particular populations, whereas the ancestral allele is not expressed after childhood (reviewed in [46]). These variants correspond to single nucleotide polymorphisms (SNPs) and several of these SNPs were shown to increase the binding of the transcription factor Oct-1 [47,48].

The “*flexible stem*” hypothesis proposes that phenotypically divergent lineages could result from genetic assimilation of alternative morphs produced by an ancestral plastic species [6]. This is observed in a few species, and extends to the genetic mechanisms generating morphological diversity [49,50]. Indeed, in a few cases, the same genes involved in the development of a particular trait show transcriptional plasticity in a plastic species and divergence of expression within or between species because of differences in their regulatory sequences. Regarding *Drosophila* pigmentation, this is the case for *t*, through *t_MSE*. This enhancer carries genetic variation involved in pigmentation divergence within or between species in the *Drosophila melanogaster* subgroup [9,11,18,36,51], and its activity is modulated by temperature [23]. We show here that the same applies to *bab* through the *bDE which* is also involved in intra- and inter-specific evolution [10]. Similarly in plants, the *Reduced Complexity* locus (*RCO*) in some species of Brassicaceae is involved in temperature modulation of leaf dissection as well as in difference of leaf dissection between species [52]. These examples suggest that the environmental sensitivity of particular genes turns them into evolutionary hotspots by facilitating the selection of the functional genetic variation they carry [50]. Indeed a given allele will produce different phenotypes in different environments. It will facilitate the selection of this allele as environmental conditions vary spatially and temporally, which increases the probability for this allele to generate a beneficial phenotype.

## Material and methods

### Fly stocks

The Canadian *Drosophila melanogaster* population from which the *Dark* and the *Pale* lines were established was kindly provided by Sam Yeaman [25]. We established each line by selecting 5–10 mated females grown at 25°C with dark or pale abdominal pigmentation. The phenotypes were fixed in 5 generations by selecting females with similar phenotype in the progeny. Each of the two lines was isogenized by brother-sister crosses for 10 generations. We used standard balancer chromosomes to construct the lines carrying the different combinations of chromosomes from the *Dark* and *Pale* lines. The *w*^*1118*^ line was the same as used in our previous studies [23]. The *t_MSE-nEGFP* transgenic line was previously described [23,36]. The *UAS-bab1-RNAi* (KK106110) and *UAS-bab2-RNAi* (GD49042) lines were from the Vienna Drosophila Resource Center [35]. The *pnr-Gal4* (BL-3039) [34] and *y-Gal4* (BL-44267) were from the Bloomington Stock Center. The line *Df(3R)C4*,p^*^/Dp(3;3)P5, *Sb*^*1*^ (BL-3071) carrying an *Abd-B* deletion and an *Abd-B* duplication was used to manipulate the dose of *Abd-B*.

### Sequencing of *tan* and *bab* regulatory sequences and transgenic reporter line constructions

The 873 bp fragments containing the *t_MSE* of the lines *Pale* (GenBank accession number: MG755262) and *Dark* (GenBank accession number: MG755261) were amplified by PCR using the following primers:

*tmf*: 5’-GATGGAAGCCGAGCACCTGGTAGA-3’
*tmR2*: 5’-TCGATAGCTACAACGTGGGTCATG-3’

The 1.5 kb fragments containing the *bab* dimorphic element (*bDE*) of the lines *Pale* (GenBank accession number: MG755259) and *Dark* (GenBank accession number: MG755258) were amplified by PCR using the following primers:

*bab1DEF*: 5’-CACATAAAAATCAGCAACAAAGTTGC-3’
*bab1DER*: 5’-CAAAACGGCGCATAAAAAGAAATTACA-3’

The 3.9 kb fragment containing the 5' *t* regulatory sequence was amplified from the *w*^*1118*^ line (GenBank accession number: MG755260) using the following primers:

*tmf2*: 5’-AAGCCGAGCACCTGGTAGAGC-3’
*tpromR3*: 5’-GTTCATTAGAGGGGCTGATGC-3’

The PCR products were cloned by topocloning in *pENTR* (InVitrogen) according to the manufacturer's instructions and sequenced (GATC Biotech). The *bDE* from the *Pale* and *Dark* lines were aligned with the sequence of *Canton S* (NCBI, EU835207.1, [21]) using Clustal Omega (ebi.ac.uk).

The *bDE* and the 5' *t* regulatory sequence were cloned by LR recombination (Gateway cloning technology) into a derivative of PH-Stinger [53] kindly provided by Dr Nicolas Gompel. The PH-Stinger vector was modified by insertion of a Gateway cloning cassette upstream of the nuclear enhanced green fluorescent protein (nEGFP). In addition, an *attB* site was inserted to allow genomic integration of transgenes using the PhiC31 integrase system [31]. After sequencing, plasmids were injected into y[1] M{vas-int.Dm}ZH-2A w[*]; M{3xP3-RFP.attP'}ZH-51C embryos (Bloomington Stock Center, BL-24482, insertion of the transgene at position 51C, BestGene Inc.).

### Genotyping of the *bab* locus

Genotyping of the *bDE* alleles was performed by PCR amplification using the following primers framing the 56 bp deletion identified in the *Dark* line:

*babDEF4*: 5’-CGGCATTAAAATTGTGTTTATGCGTGTTCG-3’
*babDER4*: 5’-TGCGAAAAGTTGTGGCTGAAATGGTAAAAT-3’

PCR amplification was performed on genomic DNA extracted from head and thorax of single flies. The abdomens were stored in ethanol, then mounted to allow pigmentation quantification. The 475 bp (*Dark* allele) and 531 bp (*Pale* allele) PCR products were separated by electrophoresis on a 1% agarose gel.

### *In situ* hybridization

*t in situ* hybridization was performed as previously reported [23].

### RT-qPCR experiments

RNA was extracted as previously described [23] from pools of 50 dissected pupae or adult female posterior abdominal epidermes (A5, A6 and A7). Morphological markers were used to collect pupae at a similar developmental stage. Three RNA replicates were analysed *per* condition for all experiments. After treatment of RNA with Turbo DNase (Ambion), cDNA were synthesized with the SuperScriptII Reverse Transcriptase kit (Invitrogen) using random primers. RT-qPCR experiments were performed in a CFX96 system using SsoFast EvaGreen SuperMix (Biorad). Expression was quantified following the Pfaffl method [54] using the geometric mean of two reference genes for normalization [55]. Reference genes were chosen with an expression level similar to the one of the tested gene: *eIF2* and *Spt6* for *bab1*, *bab2* and *Abd-B* quantification, *Act5*C and *RP49* for *t* quantification. For *Abd-B*, the primers were specific for the M isoform as the R isoform was not expressed in the pupal abdominal epidermis. Primers for *t*, *Act5C* and *RP49* were already described [23]. *bab1*, *bab2*, *Abd-B*, *eIF2* and *Spt6* primers were the followings:

*bab1F*: 5’-CAACTTGAATAAGCCCGCCG-3’
*bab1R*: 5’-CCCTCAAACGAAGGACGGAG-3’
*bab2F*: 5’-CAAAAAGCCCTTCGCCGCACTTCT-3’
*bab2R*: 5’-TGTGATGCTGCCTGCGTTGTTTGC-3’
*Abd-BF*: 5’-CGTCGCTGATGTGTGACCA-3’
*Abd-BR*: 5’-GCATTATCGTGTTGGGGCTT-3’
*eIF2F*: 5’-TCGCATCAACCTGATAGCAC-3’
*eIF2R*: 5’-ATCGTACTCGCTGGTCTTGG-3’
*Spt6F*: 5’-CGGAGGAGCTCTTCGATATG-3’
*Spt6R*: 5’-GACAGCTCTGGGAAGTCGTC-3’

### Preparation of cuticles

For pigmentation analyses, adult females between 3 and 5 days old were stored in ethanol 70% during ten days. Abdominal cuticles were cut just beyond the dorsal midline and dehydrated in ethanol 100% during 5 minutes. After dehydration, cuticles were mounted in Euparal (Roth).

For nEGFP analyses, pupal and adult abdomens were dissected in PBS, fixed 20 minutes in 3.7% paraformaldehyde in PBS, washed twice 10 minutes in PBS and mounted in Mowiol. As developmental time is sensitive to temperature, morphological markers (wing colour, location of meconium) were used to compare pupae grown at 18°C and 29°C at a similar stage of development [56].

### Image acquisition and quantification

Abdominal cuticles of adult females were imaged and quantified as previously described [23].

GFP intensity was quantified in 10 individuals for each condition. Epidermes of females expressing nEGFP under the control of *t_MSE* were imaged using a macro-apotome (Zeiss) in order to image the whole abdomen (Fig 7). Epidermes of females expressing nEGFP under the control of the 5' *t* regulatory sequence (S11 Fig) and pupae expressing nEGFP under the control of the *bDE* (Figs 4 and S6) were imaged using a micro-apotome (Zeiss). nEGFP intensity of homozygous *bDE-nEGFP* pupae was measured in hemi-segment A6 and A7 using the *ZEN* software (Zeiss) to generate maximum intensity projections of 20 z-stacks (Fig 4). For *bDE-nEGFP/+* pupae, that present a weak nEGFP signal as compared to cuticle autofluorescence, a macro was developed in ImageJ in order to extract and measure nEGFP intensity of nuclei and generate maximum intensity projections of 20 z-stacks (S6 Fig).

### Statistical analysis

One-way ANOVA and Tukey HSD tests for F2 analyses were performed using the VassarStats website (vassarstats.net). Two-way ANOVA were performed with an Excel sheet from Anastats (Anastats; http://anastats.fr). Three-way ANOVA were made using the OpenStat software (W.G. Miller, http://statprogramsplus.com/OpenStatMain.htm). We checked variance homogeneity with a Levene test and normality of residuals with a Shapiro-Wilk test (http://anastats.fr). For one-way and two-way ANOVA, when variances were not homogeneous, we performed a non-parametric ANOVA (Scheirer-Ray-Hare test) using the OpenStat software. For three-way ANOVA, when variances were not homogeneous, we transformed the data with a BoxCox transformation using R. The Eta squared of the various factors (h^2^) were calculated as SS_factor_/SS_total_ (SS: sum of squares).

## Supporting information

S1 Fig Statistics for the quantification of pigmentation in A4, A5, A6 and A7 segments of *Dark* and *Pale* females. Two-way ANOVA with full factorial models were used (Genotype, Temperature, Genotype x Temperature). df: degrees of freedom; SS: sum of squares; MS: mean squares; F: F-statistic; p: p-value.
(DOCX)

S2 Fig ANOVA tables for pigmentation of A4, A5, A6 and A7 segments of the lines bearing the 8 different combinations of chromosomes from the *Dark* and the *Pale* lines. Three-way ANOVA with full factorial model were used (Chromosomes X, II, III and all interactions). df: degrees of freedom; SS: sum of squares; MS: mean squares; F: F-statistic; p: p-value. h^2^: Eta squared.
(DOCX)

S3 Fig Gel-electrophoresis showing the PCR products obtained from *bab* genotyping of the 40 F2 individuals from an initial *Dark* x *Pale* cross. The *bDE*^*D*^ and *bDE*^*P*^ alleles differ by a 56 bp deletion. D, P and F1: control amplification on genomic DNA from *Dark*, *Pale* and *F1* individuals, respectively. NC: negative control. MWM: molecular weight marker.
(TIF)

S4 Fig One-way ANOVA for quantification of pigmentation in A5, A6 and A7 segments of 40 F2 individuals from an initial *Dark* x *Pale* cross. df: degrees of freedom; SS: sum of squares; MS: mean squares; F: F-statistic; p: p-value. h^2^: Eta squared.
(DOCX)

S5 Fig Statistics for the quantification of nEGFP expression in A6 and A7 segments of *bDE*^*D*^*-nEGFP* and *bDE*^*P*^*-nEGFP* transgenic lines. A6: Non-parametric ANOVA (Sheirer-Ray-Hare test). df: degrees of freedom; SS: sum of squares; MS: mean squares; F: F-statistic; P>F: p-value for P>F; H: chi-square statistic; p>H: p-value of the Scheirer-Ray-Hare test. A7: Two-way ANOVA. df: degrees of freedom; SS: sum of squares; MS: mean squares; F: F-statistic; p: p-value. h^2^: Eta squared.
(DOCX)

S6 Fig Activity of *bDE* from the *Dark* or *Pale* lines in different conditions of temperature and *Abd-B* dose. Intensity of nEGFP in *bDE*^*D*^*-nEGFP* and *bDE*^*P*^*-nEGFP* heterozygous transgenic lines, at 18°C or 29°C, with 1 dose, 2 doses or 3 doses of *Abd-B*. A6 and A7 segments are delimited with white dashed lines. G: genitalia.
(TIF)

S7 Fig Statistics for the quantification of nEGFP expression in segments A6 and A7 of *bDE*^*D*^*-nEGFP* and *bDE*^*P*^*-nEGFP* transgenic lines. For A6 and A7, three-way ANOVA were performed on Box-Cox transformed measures of nEGFP intensities extracted from nEGFP positive nuclei. G: genotype (allele *bDE*^*D*^ or *bDE*^*P*^); D: dose of *Abd-B* (1, 2 or 3); T: temperature (18°C or 29°C). df: degrees of freedom; SS: sum of squares; MS: mean squares; F: F-statistic; p: p-value; h^2^: Eta squared.
(DOCX)

S8 Fig *Abd-B* expression in the pupal posterior abdominal epidermis does not vary with temperature. RT-qPCR quantification of *Abd-B* expression in the posterior abdominal epidermis (segments A5, A6 and A7) from female pupae of the *Dark* and *Pale* lines raised at 18°C or 29°C. The expression of *Abd-B* was normalized with the geometric mean of *eIF2* and *Spt6* expression. Error bars represent the standard deviation (3 replicates of 50 individuals *per* condition). Statistics: t-tests. NS: non significant.
(TIF)

S9 Fig Statistics for RT-qPCR quantification of *bab1* and *bab2* expression in pupal epidermis of females with one or two doses of *Abd-B*, grown at 18°C and 29°C. Two-way ANOVA. df: degrees of freedom; SS: sum of squares; MS: mean squares; F: F-statistic; p: p-value; h^2^: Eta squared.
(DOCX)

S10 Fig Statistics for RT-qPCR quantification of *bab1* and *bab2* abdominal expression in *Pale* and *Dark* lines (pupae and adults, at 18°C and 29°C). Two-way ANOVA or non-parametric two-way ANOVA (Scheirer-Ray-Hare test). df: degrees of freedom; SS: sum of squares; MS: mean squares; F: F-statistic; p: p-value. h^2^: Eta squared. Scheirer-Ray-Hare test: df: degrees of freedom; SS: sum of squares; MS: mean squares; F: F-statistic; P>F p-value; H: chi-square statistic; p>H: p-value of the Scheirer-Ray-Hare test.
(DOCX)

S11 Fig Down-regulation of *bab1* and *bab2* in the abdominal epidermis increases the activity of the 5' *t* regulatory sequence. A: Cuticles of control females (left) and females in which *bab1* and *bab2* were down-regulated using *UAS-RNAi* transgenes and the *y-Gal4* driver (right). *bab* down-regulation induces an increase of melanin production in the posterior segments A5, A6, A7.
B. Effect of *bab1* and *bab2* down-regulation on 5' *t* regulatory region activity (5'*t-nEGFP* transgenic line). Compared to the control (left), *bab* down-regulation (right) increase *nEGFP* expression driven by the 5' *t* regulatory region. In A and B, crosses were performed at 25°C.
(TIF)

S12 Fig Statistics for the quantification of *t* abdominal expression by RT-qPCR in *Pale* and *Dark* females (18°C and 29°C). Two-way ANOVA. df: degrees of freedom; SS: sum of squares; MS: mean squares; F: F-statistic; p: p-value. h^2^: Eta squared.
(DOCX)

S1 Dataset Raw data for Fig 1. Values of pigmentation intensities in A4, A5, A6 and A7 segments of *Pale* and *Dark* females at 18°C, 25°C and 29°C (n = 10 *per* condition). Nomenclature of the individuals: name of the line (D/P), temperature (18/25/29), number (1 to 10).
(XLSX)

S2 Dataset Raw data for Fig 2. Values of pigmentation intensities in A4, A5, A6 and A7 segments of females with the 8 combinations of chromosomes X, II and III from the *Dark* and *Pale* lines (n = 10 *per* condition). Each individual is named according to its chromosomal combination (D or P) and number (1 to 10).
(XLSX)

S3 Dataset Raw data for Fig 3. Values of pigmentation intensities in A5, A6 and A7 segments of 40 F2 females from an initial cross between *Dark* females and *Pale* males. For each individual, its genotype at the *bab* locus is indicated.
(XLSX)

S4 Dataset Raw data for Fig 4. Values of nEGFP intensity in A6 and A7 segments of transgenic *bDE*^*D*^*-nEGFP* and *bDE*^*P*^*-nEGFP* female pharates grown at 18°C or 29°C (n = 10 *per* condition).
(XLSX)

S5 Dataset Raw data for Fig 5. Values of nEGFP intensity extracted from nEGFP positive nuclei in A6 (Int-A6) and A7 (Int-A7) segments of *bDE*^*D*^*-nEGFP* (D) or *bDE*^*P*^*-nEGFP* (P) female pharates with 1, 2 or 3 doses of *Abd-B*, and grown at 18°C or 29°C (n = 10 *per* condition). nEGFP intensities were treated with a Box-Cox transformation (Int-A6-BC and Int-A7-BC).
(XLSX)

S6 Dataset Raw data for S8 Fig. Values of *Abd-B* expression (normalized with the geometric mean of *eIF2* and *Spt6* expressions) in posterior pupal abdomimal epidermis of *Pale* and *Dark* females grown at 18°C or 29°C (3 replicates *per* condition).
(XLSX)

S7 Dataset Raw data for Fig 6. Values of *bab1* and *bab2* expressions (normalized with the geometric mean of *eIF2* and *Spt6* expressions) in posterior pupal abdominal epidermis of females with one or two doses of *Abd-B* grown at 18°C or 29°C (3 replicates *per* condition).
(XLSX)

S8 Dataset Raw data for Fig 7. Values of *bab1* and *bab2* expressions (normalized with the geometric mean of *eIF2* and *Spt6* expressions) in pupal or adult posterior abdominal epidermis of *Dark* and *Pale* females grown at 18°C or 29°C (3 replicates *per* condition).
(XLSX)

S9 Dataset Raw data for Fig 9. Values of *tan* expression (normalized with the geometric mean of *Act5c* and *RP49* expressions) in the posterior abdominal epidermis of *Dark* and *Pale* young females grown at 18°C and 29°C (3 replicates *per* condition).
(XLSX)
