# Supplementary material for: bric à brac (bab), a central player in the gene regulatory network that mediates thermal plasticity of pigmentation in Drosophila melanogaster
Source: PLoS Genet. 2018 Aug 1;14(8):e1007573. doi: 10.1371/journal.pgen.1007573 (PMC6089454; doi:10.1371/journal.pgen.1007573)
Supplement: S1 Fig — Two-way ANOVA with full factorial models were used (Genotype, Temperature, Genotype x Temperature). df: degrees of freedom; SS: sum of squares; MS: mean squares; F: F-statistic; p: p-value. (DOCX) [file pgen.1007573.s001.docx]

A4

|  | df | SS | MS | F | p |
| --- | --- | --- | --- | --- | --- |
| G | 1 | 167.14 | 167.14 | 4.879 | 0.03145 |
| T | 2 | 1621.78 | 810.89 | 23.670 | 0.00000 |
| GxT | 2 | 171.88 | 85.94 | 2.509 | 0.09083 |
| Residuals | 54 | 1849.93 | 34.26 |  |  |
| Total | 59 | 3810.73 |  |  |  |

A5

|  | df | SS | MS | F | p |
| --- | --- | --- | --- | --- | --- |
| G | 1 | 997.38 | 997.38 | 31.621 | 0.00000 |
| T | 2 | 3148.68 | 1574.34 | 49.913 | 0.00000 |
| GxT | 2 | 84.93 | 42.46 | 1.346 | 0.26879 |
| Residuals | 54 | 1703.25 | 31.54 |  |  |
| Total | 59 | 5934.23 |  |  |  |

A6

|  | df | SS | MS | F | p |
| --- | --- | --- | --- | --- | --- |
| G | 1 | 25242.11 | 25242.11 | 567.514 | 0.00000 |
| T | 2 | 13561.15 | 6780.58 | 152.447 | 0.00000 |
| GxT | 2 | 1717.61 | 858.81 | 19.308 | 0.00000 |
| Residuals | 54 | 2401.83 | 44.48 |  |  |
| Total | 59 | 42922.71 |  |  |  |

A7

|  | df | SS | MS | F | p |
| --- | --- | --- | --- | --- | --- |
| G | 1 | 30262.16 | 30262.16 | 750.222 | 0.00000 |
| T | 2 | 17184.56 | 8592.28 | 213.009 | 0.00000 |
| GxT | 2 | 1928.83 | 964.41 | 23.909 | 0.00000 |
| Residuals | 54 | 2178.23 | 40.34 |  |  |
| Total | 59 | 51553.77 |  |  |  |
