# Supplementary material for: bric à brac (bab), a central player in the gene regulatory network that mediates thermal plasticity of pigmentation in Drosophila melanogaster
Source: PLoS Genet. 2018 Aug 1;14(8):e1007573. doi: 10.1371/journal.pgen.1007573 (PMC6089454; doi:10.1371/journal.pgen.1007573)
Supplement: S2 Fig — Three-way ANOVA with full factorial model were used (Chromosomes X, II, III and all interactions). df: degrees of freedom; SS: sum of squares; MS: mean squares; F: F-statistic; p: p-value. h2: Eta squared. (DOCX) [file pgen.1007573.s002.docx]

A4

|  | df | SS | MS | F | p | h^2^ |
| --- | --- | --- | --- | --- | --- | --- |
| X | 1 | 14.543 | 14.543 | 0.131 | 0.719 | 0.001 |
| II | 1 | 299.565 | 299.565 | 2.697 | 0.105 | 0.028 |
| III | 1 | 1432.964 | 1432.964 | 12.903 | 0.001 | 0.136 |
| XxII | 1 | 286.384 | 286.384 | 2.579 | 0.113 | 0.027 |
| XxIII | 1 | 175.513 | 175.513 | 1.580 | 0.213 | 0.016 |
| IIxIII | 1 | 114.704 | 114.704 | 1.033 | 0.313 | 0.011 |
| XxIIxIII | 1 | 213.384 | 213.384 | 1.921 | 0.170 | 0.020 |
| Residuals | 72 | 7995.928 | 111.055 |  |  | 0.759 |
| Total | 79 | 10532.985 | 133.329 |  |  |  |

A5

|  | df | SS | MS | F | p | h^2^ |
| --- | --- | --- | --- | --- | --- | --- |
| X | 1 | 15.412 | 15.412 | 0.165 | 0.686 | 0.001 |
| II | 1 | 408.066 | 408.066 | 4.368 | 0.040 | 0.038 |
| III | 1 | 3112.912 | 3112.912 | 33.323 | <0.001 | 0.294 |
| XxII | 1 | 85.549 | 85.549 | 0.916 | 0.342 | 0.008 |
| XxIII | 1 | 23.224 | 23.224 | 0.249 | 0.620 | 0.002 |
| IIxIII | 1 | 66.565 | 66.565 | 0.713 | 0.401 | 0.006 |
| XxIIxIII | 1 | 137.147 | 137.147 | 1.468 | 0.230 | 0.013 |
| Residuals | 72 | 6725.924 | 93.416 |  |  | 0.636 |
| Total | 79 | 10574.799 | 133.858 |  |  |  |

A6

|  | df | SS | MS | F | p | h^2^ |
| --- | --- | --- | --- | --- | --- | --- |
| X | 1 | 27.489 | 27.489 | 0.340 | 0.562 | 7.744E-04 |
| II | 1 | 2383.803 | 2383.803 | 29.463 | <0.001 | 0.067 |
| III | 1 | 27200.365 | 27200.365 | 336.190 | <0.001 | 0.700 |
| XxII | 1 | 24.552 | 24.552 | 0.303 | 0.583 | 6.916E-04 |
| XxIII | 1 | 0.019 | 0.019 | 0.000 | 0.988 | 5.352E-07 |
| IIxIII | 1 | 14.546 | 14.546 | 0.180 | 0.673 | 4.097E-04 |
| XxIIxIII | 1 | 20.559 | 20.559 | 0.254 | 0.616 | 5.791E-04 |
| Residuals | 72 | 5825.348 | 80.908 |  |  | 0.164 |
| Total | 79 | 35496.681 | 449.325 |  |  |  |

A7

|  | df | SS | MS | F | p | h^2^ |
| --- | --- | --- | --- | --- | --- | --- |
| X | 1 | 312.544 | 312.544 | 5.799 | 0.019 | 0.007 |
| II | 1 | 1971.036 | 1971.036 | 36.572 | <0.001 | 0.044 |
| III | 1 | 36921.758 | 36921.758 | 685.081 | <0.001 | 0.832 |
| XxII | 1 | 123.154 | 123.154 | 2.285 | 0.135 | 0.003 |
| XxIII | 1 | 0.133 | 0.133 | 0.002 | 0.961 | 2.999E-06 |
| IIxIII | 1 | 1049.750 | 1049.750 | 19.478 | <0.001 | 0.023 |
| XxIIxIII | 1 | 79.459 | 79.459 | 1.474 | 0.229 | 0.008 |
| Residuals | 72 | 3880.367 | 53.894 |  |  | 0.088 |
| Total | 79 | 44338.201 | 561.243 |  |  |  |
