# Supplementary figures and images for: bric à brac (bab), a central player in the gene regulatory network that mediates thermal plasticity of pigmentation in Drosophila melanogaster
Source: PLoS Genet. 2018 Aug 1;14(8):e1007573. doi: 10.1371/journal.pgen.1007573 (PMC6089454; doi:10.1371/journal.pgen.1007573)

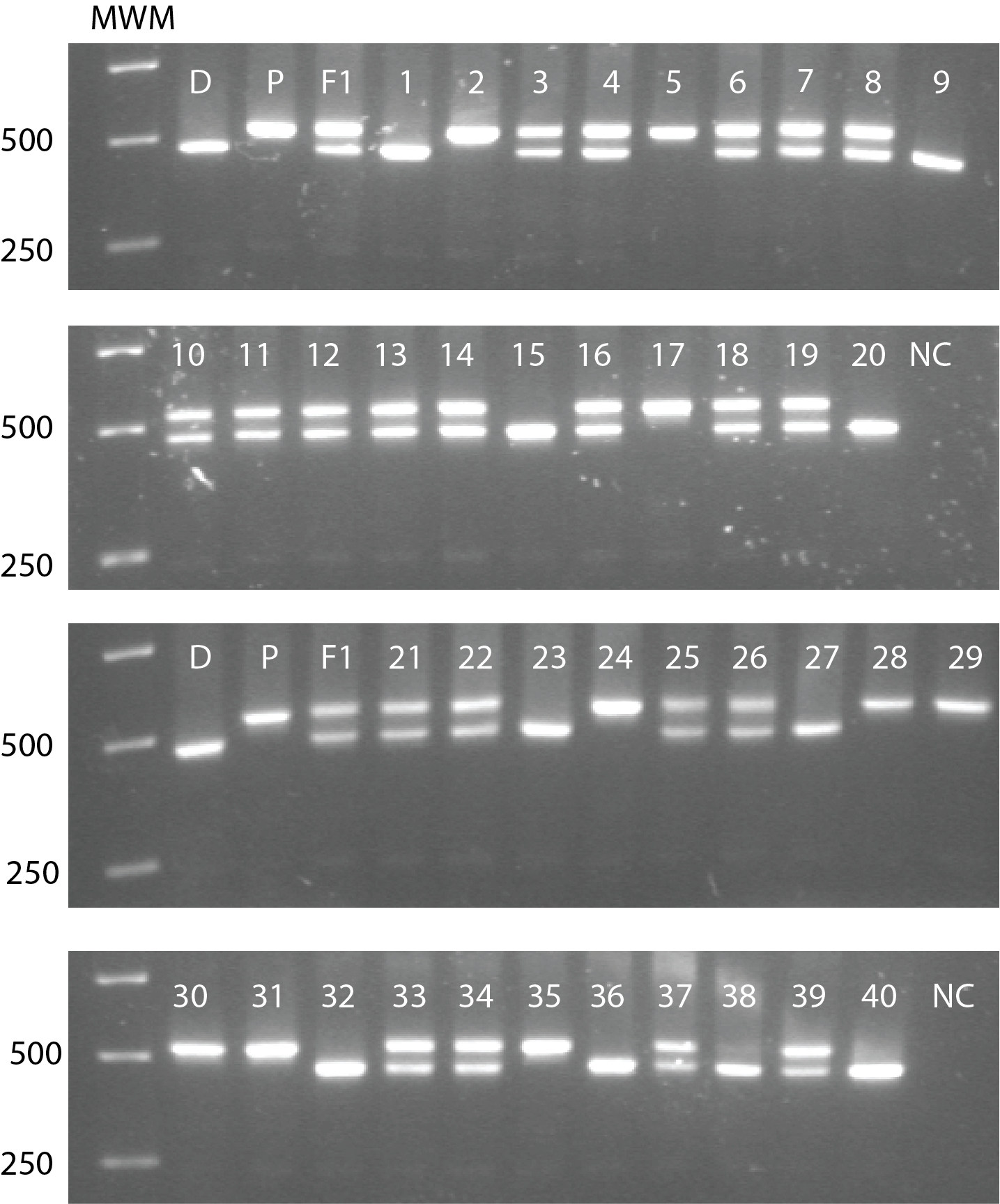

Supplement: S3 Fig — The bDED and bDEP alleles differ by a 56 bp deletion. D, P and F1: control amplification on genomic DNA from Dark, Pale and F1 individuals, respectively. NC: negative control. MWM: molecular weight marker. (TIF) [file pgen.1007573.s003.tif]

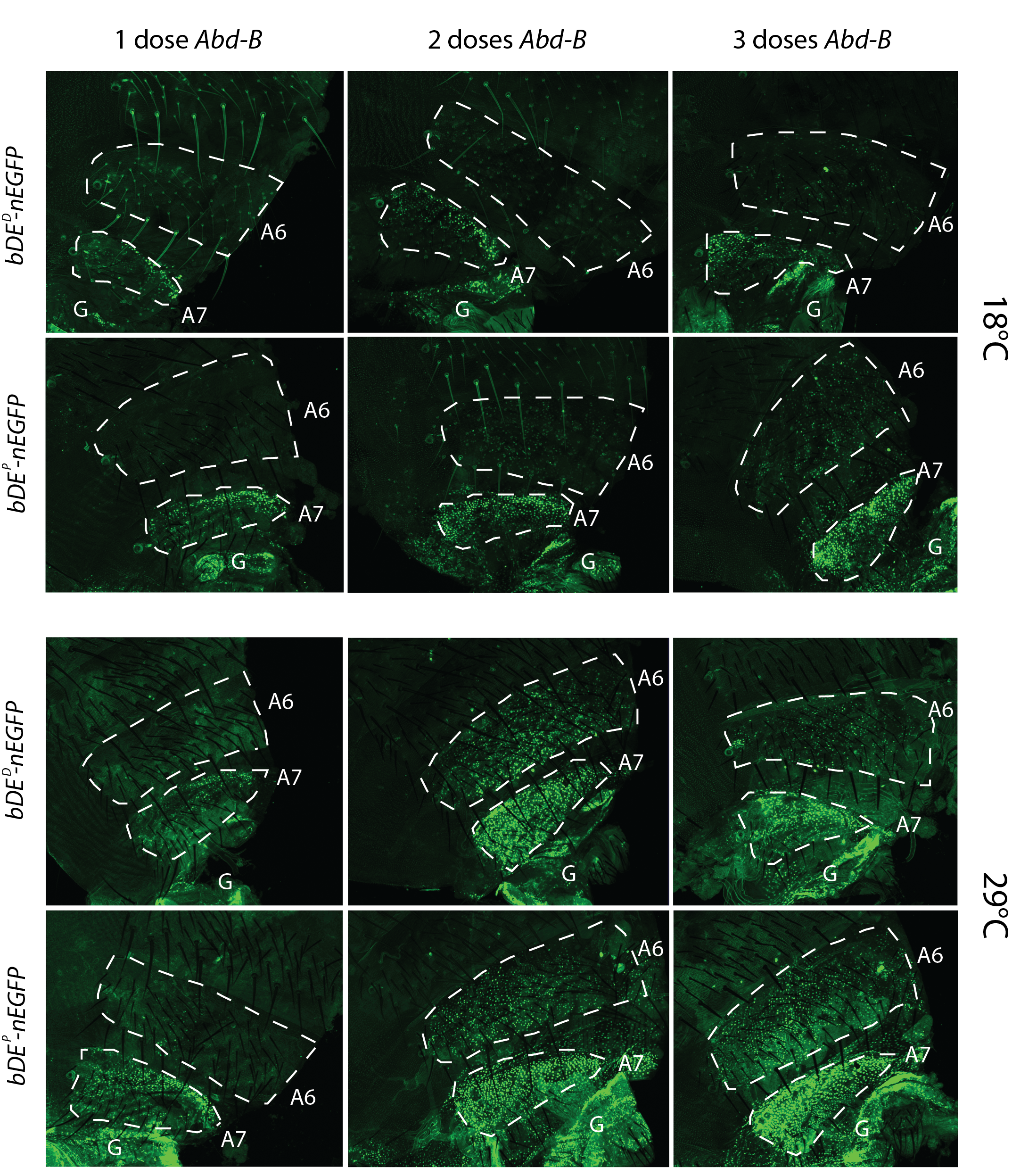

Supplement: S6 Fig — Intensity of nEGFP in bDED-nEGFP and bDEP-nEGFP heterozygous transgenic lines, at 18°C or 29°C, with 1 dose, 2 doses or 3 doses of Abd-B. A6 and A7 segments are delimited with white dashed lines. G: genitalia. (TIF) [file pgen.1007573.s006.tif]

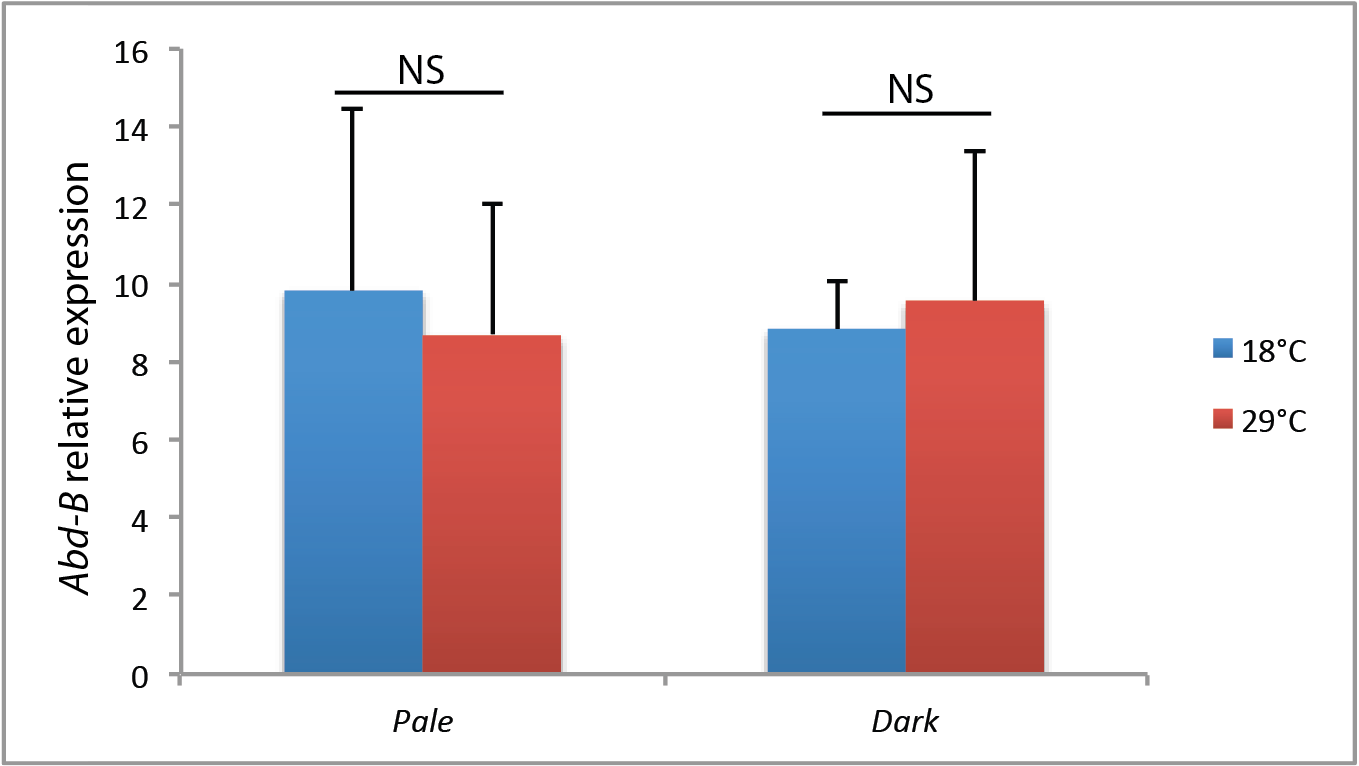

Supplement: S8 Fig — RT-qPCR quantification of Abd-B expression in the posterior abdominal epidermis (segments A5, A6 and A7) from female pupae of the Dark and Pale lines raised at 18°C or 29°C. The expression of Abd-B was normalized with the geometric mean of eIF2 and Spt6 expression. Error bars represent the standard deviation (3 replicates of 50 individuals per condition). Statistics: t-tests. NS: non significant. (TIF) [file pgen.1007573.s008.tif]

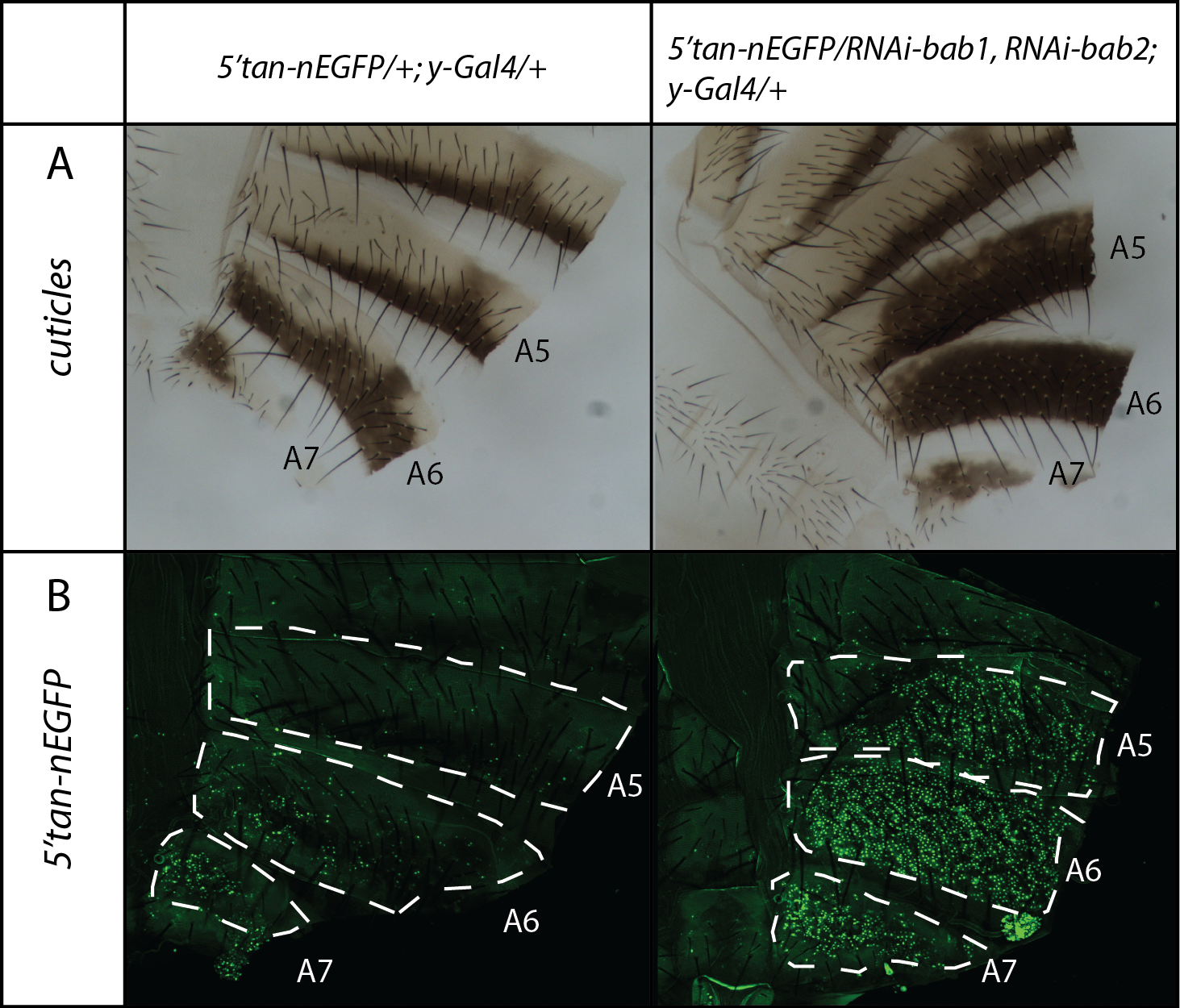

Supplement: S11 Fig — A: Cuticles of control females (left) and females in which bab1 and bab2 were down-regulated using UAS-RNAi transgenes and the y-Gal4 driver (right). bab down-regulation induces an increase of melanin production in the posterior segments A5, A6, A7. B. Effect of bab1 and bab2 down-regulation on 5' t regulatory region activity (5't-nEGFP transgenic line). Compared to the control (left), bab down-regulation (right) increase nEGFP expression driven by the 5' t regulatory region. In A and B, crosses were performed at 25°C. (TIF) [file pgen.1007573.s011.tif]
