# Supplementary material for: bric à brac (bab), a central player in the gene regulatory network that mediates thermal plasticity of pigmentation in Drosophila melanogaster
Source: PLoS Genet. 2018 Aug 1;14(8):e1007573. doi: 10.1371/journal.pgen.1007573 (PMC6089454; doi:10.1371/journal.pgen.1007573)
Supplement: S4 Fig — df: degrees of freedom; SS: sum of squares; MS: mean squares; F: F-statistic; p: p-value. h2: Eta squared. (DOCX) [file pgen.1007573.s004.docx]

A5

|  | df | SS | MS | F | p | h^2^ |
| --- | --- | --- | --- | --- | --- | --- |
| Genotype | 2 | 108.3246 | 54.1623 | 0.73 | 0.488720 | 0.038102 |
| Residuals | 37 | 2734.6864 | 73.9104 |  |  |  |
| Total | 39 | 2843.011 |  |  |  |  |

A6

|  | df | SS | MS | F | p | h^2^ |
| --- | --- | --- | --- | --- | --- | --- |
| Genotype | 2 | 9397.5526 | 4698.7763 | 29.39 | <0.001*** | 0.6137264 |
| Residuals | 37 | 59.14729 | 159.8575 |  |  |  |
| Total | 39 | 15312.2816 |  |  |  |  |

A7

|  | df | SS | MS | F | p | h^2^ |
| --- | --- | --- | --- | --- | --- | --- |
| Genotype | 2 | 11968.5102 | 5984.2551 | 52.06 | <0.001*** | 0.7378193 |
| Residuals | 37 | 4252.9546 | 114.9447 |  |  |  |
| Total | 39 | 16221.4648 |  |  |  |  |
