# Supplementary material for: bric à brac (bab), a central player in the gene regulatory network that mediates thermal plasticity of pigmentation in Drosophila melanogaster
Source: PLoS Genet. 2018 Aug 1;14(8):e1007573. doi: 10.1371/journal.pgen.1007573 (PMC6089454; doi:10.1371/journal.pgen.1007573)
Supplement: S5 Fig — A6: Non-parametric ANOVA (Sheirer-Ray-Hare test). df: degrees of freedom; SS: sum of squares; MS: mean squares; F: F-statistic; P>F: p-value for P>F; H: chi-square statistic; p>H: p-value of the Scheirer-Ray-Hare test. A7: Two-way ANOVA. df: degrees of freedom; SS: sum of squares; MS: mean squares; F: F-statistic; p: p-value. h2: Eta squared. (DOCX) [file pgen.1007573.s005.docx]

A6 (Scheirer-Ray-Hare test)

|  | df | SS | MS | F | P>F | H | p>H |
| --- | --- | --- | --- | --- | --- | --- | --- |
| G | 1 | 115.600 | 115.600 | 2.145 | 0.152 | 0.846 | 0.358 |
| T | 1 | 2890.000 | 2890.000 | 53.629 | 0.000 | 21.146 | <0.001 |
| GxT | 1 | 384.400 | 384.400 | 7.133 | 0.011 | 2.813 | 0.094 |
| Residuals | 36 | 1940.000 | 53.889 |  |  |  |  |
| Total | 39 | 5330.000 | 136.667 |  |  |  |  |

A7 (two-way ANOVA)

|  | df | SS | MS | F | p | h^2^ |
| --- | --- | --- | --- | --- | --- | --- |
| G | 1 | 9955690.19 | 9955690.19 | 22.710 | <0.001 | 0.158 |
| T | 1 | 35856503.29 | 35856503.29 | 81.792 | <0.001 | 0.568 |
| GxT | 1 | 1498993.31 | 1498993.31 | 3.419 | 0.0727 | 0.024 |
| Residuals | 36 | 15781891.22 | 438385.87 |  |  | 0.250 |
| Total | 39 | 63093078.01 |  |  |  |  |
