# Supplementary material for: bric à brac (bab), a central player in the gene regulatory network that mediates thermal plasticity of pigmentation in Drosophila melanogaster
Source: PLoS Genet. 2018 Aug 1;14(8):e1007573. doi: 10.1371/journal.pgen.1007573 (PMC6089454; doi:10.1371/journal.pgen.1007573)
Supplement: S7 Fig — For A6 and A7, three-way ANOVA were performed on Box-Cox transformed measures of nEGFP intensities extracted from nEGFP positive nuclei. G: genotype (allele bDED or bDEP); D: dose of Abd-B (1, 2 or 3); T: temperature (18°C or 29°C). df: degrees of freedom; SS: sum of squares; MS: mean squares; F: F-statistic; p: p-value; h2: Eta squared. (DOCX) [file pgen.1007573.s007.docx]

A6

|  | df | SS | MS | F | p | h^2^ |
| --- | --- | --- | --- | --- | --- | --- |
| G | 1 | 653 | 653 | 7.2130 | 0.008 | 0.008 |
| D | 2 | 41902 | 20951 | 231.5352 | 0.000 | 0.540 |
| T | 1 | 18270 | 18270 | 201.9097 | 0.000 | 0.235 |
| GxD | 2 | 1598 | 799 | 8.8298 | 0.000 | 0.021 |
| GxT | 1 | 1670 | 1670 | 18.4513 | 0.000 | 0.021 |
| DxT | 2 | 2960 | 1480 | 16.3549 | 0.000 | 0.038 |
| GxDxT | 2 | 780 | 390 | 4.3090 | 0.016 | 0.010 |
| Residuals | 108 | 9773 | 90.49 |  |  | 0.126 |
| Total | 119 | 77606 | 652.15 |  |  |  |

A7

|  | df | SS | MS | F | p | h^2^ |
| --- | --- | --- | --- | --- | --- | --- |
| G | 1 | 52350 | 52350 | 83.6074 | 0.000 | 0.147 |
| D | 2 | 68667 | 34333.5 | 54.8335 | 0.000 | 0.193 |
| T | 1 | 140027 | 140027 | 223.6336 | 0.000 | 0.394 |
| GxD | 2 | 8562 | 4281 | 6.8371 | 0.002 | 0.024 |
| GxT | 1 | 1791 | 1791 | 2.8609 | 0.094 | 0.005 |
| DxT | 2 | 12040 | 6020 | 9.61 | 0.000 | 0.034 |
| GxDxT | 2 | 4397 | 2198.5 | 3.51 | 0.033 | 0.012 |
| Residuals | 108 | 67624 | 626.15 |  |  | 0.190 |
| Total | 119 | 355458 | 2987.04 |  |  |  |
