# Supplementary material for: bric à brac (bab), a central player in the gene regulatory network that mediates thermal plasticity of pigmentation in Drosophila melanogaster
Source: PLoS Genet. 2018 Aug 1;14(8):e1007573. doi: 10.1371/journal.pgen.1007573 (PMC6089454; doi:10.1371/journal.pgen.1007573)
Supplement: S9 Fig — Two-way ANOVA. df: degrees of freedom; SS: sum of squares; MS: mean squares; F: F-statistic; p: p-value; h2: Eta squared. (DOCX) [file pgen.1007573.s009.docx]

*bab1*

|  | df | SS | MS | F | p | h^2^ |
| --- | --- | --- | --- | --- | --- | --- |
| T | 1 | 0.26 | 0.26 | 8.691 | 0.01848 | 0.072 |
| D | 1 | 2.82 | 2.82 | 95.929 | 0.00001 | 0.792 |
| DxT | 1 | 0.25 | 0.25 | 8.478 | 0.01954 | 0.070 |
| Residuals | 8 | 0.24 | 0.03 |  |  |  |
| Total | 11 | 3.56 |  |  |  |  |

*bab2*

|  | df | SS | MS | F | p | h^2^ |
| --- | --- | --- | --- | --- | --- | --- |
| T | 1 | 7.93 | 7.93 | 7.393 | 0.02629 | 0.230 |
| D | 1 | 16.57 | 16.57 | 15.445 | 0.00436 | 0.480 |
| DxT | 1 | 1.41 | 1.41 | 1.314 | 0.28472 |  |
| Residuals | 8 | 8.58 | 1.07 |  |  |  |
| Total | 11 | 34.50 |  |  |  |  |
