# Supplementary material for: bric à brac (bab), a central player in the gene regulatory network that mediates thermal plasticity of pigmentation in Drosophila melanogaster
Source: PLoS Genet. 2018 Aug 1;14(8):e1007573. doi: 10.1371/journal.pgen.1007573 (PMC6089454; doi:10.1371/journal.pgen.1007573)
Supplement: S10 Fig — Two-way ANOVA or non-parametric two-way ANOVA (Scheirer-Ray-Hare test). df: degrees of freedom; SS: sum of squares; MS: mean squares; F: F-statistic; p: p-value. h2: Eta squared. Scheirer-Ray-Hare test: df: degrees of freedom; SS: sum of squares; MS: mean squares; F: F-statistic; P>F p-value; H: chi-square statistic; p>H: p-value of the Scheirer-Ray-Hare test. (DOCX) [file pgen.1007573.s010.docx]

*bab1* pupae, ANOVA

|  | df | SS | MS | F | p | h^2^ |
| --- | --- | --- | --- | --- | --- | --- |
| Genotype | 1 | 0.96 | 0.96 | 18.747 | 0.00251 | 0.691 |
| Temperature | 1 | 0.01 | 0.01 | 0.210 | 0.65897 | 0.007 |
| GxT | 1 | 0.02 | 0.02 | 0.308 | 0.59400 | 0.014 |
| Residuals | 8 | 0.41 | 0.05 |  |  | 0.295 |
| Total | 11 | 1.39 |  |  |  |  |

*bab2* pupae, ANOVA

|  | df | SS | MS | F | p | h^2^ |
| --- | --- | --- | --- | --- | --- | --- |
| Genotype | 1 | 0.78 | 0.78 | 0.750 | 0.41173 | 0.064 |
| Temperature | 1 | 3.00 | 3.00 | 2.885 | 0.12784 | 0.248 |
| GxT | 1 | 0.03 | 0.03 | 0.025 | 0.87883 | 0.002 |
| Residuals | 8 | 8.31 | 1.04 |  |  | 0.083 |
| Total | 11 | 12.11 |  |  |  |  |

*bab1* adults, Scheirer-Ray-Hare test

|  | df | SS | MS | F | p>F | H | p>H |
| --- | --- | --- | --- | --- | --- | --- | --- |
| Genotype | 1 | 48.000 | 48.000 | 10.286 | 0.012 | 3.692 | 0.055 |
| Temperature | 1 | 56.333 | 56.333 | 12.071 | 0.008 | 4.333 | 0.037 |
| GxT | 1 | 1.333 | 1.333 | 0.286 | 0.608 | 0.103 | 0.749 |
| Residuals | 8 | 37.333 | 4.667 |  |  |  |  |
| Total | 11 | 143.000 | 13.000 |  |  |  |  |

*bab2* adults, ANOVA

|  | df | SS | MS | F | p | h^2^ |
| --- | --- | --- | --- | --- | --- | --- |
| Genotype | 1 | 6.98 | 6.98 | 14.397 | 0.00528 | 0.429 |
| Temperature | 1 | 4.94 | 4.94 | 10.181 | 0.01279 | 0.303 |
| GxT | 1 | 0.48 | 0.48 | 0.997 | 0.34718 | 0.029 |
| Residuals | 8 | 3.88 | 0.48 |  |  | 0.238 |
| Total | 11 | 16.28 |  |  |  |  |
