# Supplementary material for: bric à brac (bab), a central player in the gene regulatory network that mediates thermal plasticity of pigmentation in Drosophila melanogaster
Source: PLoS Genet. 2018 Aug 1;14(8):e1007573. doi: 10.1371/journal.pgen.1007573 (PMC6089454; doi:10.1371/journal.pgen.1007573)
Supplement: S12 Fig — Two-way ANOVA. df: degrees of freedom; SS: sum of squares; MS: mean squares; F: F-statistic; p: p-value. h2: Eta squared. (DOCX) [file pgen.1007573.s012.docx]

ANOVA

|  | df | SS | MS | F | p | h^2^ |
| --- | --- | --- | --- | --- | --- | --- |
| G | 1 | 0.32 | 0.32 | 13.485 | 0.00629 | 0.068 |
| T | 1 | 4.10 | 4.10 | 172.519 | 0.00000 | 0.870 |
| GxT | 1 | 0.10 | 0.10 | 4.027 | 0.07969 | 0.021 |
| Residuals | 8 | 0.19 | 0.02 |  |  |  |
| Total | 11 | 4.71 |  |  |  |  |
